# Genetic Dissection of Photoperiod Response Based on GWAS of Pre-Anthesis Phase Duration in Spring Barley

**DOI:** 10.1371/journal.pone.0113120

**Published:** 2014-11-24

**Authors:** Ahmad M. Alqudah, Rajiv Sharma, Raj K. Pasam, Andreas Graner, Benjamin Kilian, Thorsten Schnurbusch

**Affiliations:** 1 Research group Plant Architecture, Leibniz Institute of Plant Genetics and Crop Plant Research (IPK), Corrensstrasse 3, OT Gatersleben, D-06466 Stadt Seeland, Germany; 2 Research group Genome Diversity, Genebank Department, Leibniz Institute of Plant Genetics and Crop Plant Research (IPK), Corrensstrasse 3, OT Gatersleben, D-06466 Stadt Seeland, Germany; Department of Agriculture and Food Western Australia, Australia

## Abstract

Heading time is a complex trait, and natural variation in photoperiod responses is a major factor controlling time to heading, adaptation and grain yield. In barley, previous heading time studies have been mainly conducted under field conditions to measure total days to heading. We followed a novel approach and studied the natural variation of time to heading in a world-wide spring barley collection (218 accessions), comprising of 95 photoperiod-sensitive (*Ppd-H1*) and 123 accessions with reduced photoperiod sensitivity (*ppd-H1*) to long-day (LD) through dissecting pre-anthesis development into four major stages and sub-phases. The study was conducted under greenhouse (GH) conditions (LD; 16/8 h; ∼20/∼16°C day/night). Genotyping was performed using a genome-wide high density 9K single nucleotide polymorphisms (SNPs) chip which assayed 7842 SNPs. We used the barley physical map to identify candidate genes underlying genome-wide association scans (GWAS). GWAS for pre-anthesis stages/sub-phases in each photoperiod group provided great power for partitioning genetic effects on floral initiation and heading time. In addition to major genes known to regulate heading time under field conditions, several novel QTL with medium to high effects, including new QTL having major effects on developmental stages/sub-phases were found to be associated in this study. For example, highly associated SNPs tagged the physical regions around *HvCO1* (barley *CONSTANS1*) and *BFL* (*BARLEY FLORICAULA*/*LEAFY*) genes. Based upon our GWAS analysis, we propose a new genetic network model for each photoperiod group, which includes several newly identified genes, such as several *HvCO-like* genes, belonging to different heading time pathways in barley.

## Introduction

Heading time is an important trait for barley (*Hordeum vulgare* L.) adapting to particular environmental cues and hence for maximizing grain yield. Its complex genetic architecture was considered as one of the major breeding goals during last century. The capacity to regulate heading time provides crop plants with the opportunity to successfully complete their life cycle under a wide range of environments, which exceed the distribution range of their wild relatives [1]. The optimal time to flower is crucial for high crop grain yields [2]. For decades, studies on heading/flowering time solely focused on the total number of days until heading/flowering and its effect on grain yield in response to environmental cues. However, the time prior to anthesis (i.e. pre-anthesis developmental phases) in barley consists of vegetative, early and late reproductive phases [3], [4]. Grain yield and yield potential are significantly influenced by the reproductive, pre-anthesis phase durations [5], which were shown to be genetically controlled [3]. Despite the importance of this earlier phase of development, most of what we know about the genetic control of pre-anthesis phases is purely based on traditional quantitative trait locus (QTL) analysis, wherein often vegetative and reproductive phases were not clearly separable [6], [7]. Alqudah and Schnurbusch [3] proposed an amended approach for dissecting the longest pre-anthesis phase (late-reproductive phase) into three sub-phases: awn primordium (AP) to tipping (TIP); TIP to heading (HD); HD to anther extrusion (AE). This refined approach, based upon clearly defined developmental pre-anthesis stages, might shed more light on the causal genetic factors responsible for the variation in developmental stages/sub-phases in response to photoperiod in barley.

Most studies in barley aimed to unravel the genetics of heading time and the underlying specific genes in response to photoperiod, vernalization and/or earliness per se. The first two factors change heading time in response to environmental conditions, while the last factor determines heading time independent of photoperiod and temperature [8]. In barley, a long-day (LD) crop, the *PSEUDO-RESPONSE REGULATOR* (*HvPRR37*) gene, also known as *PHOTOPERIOD RESPONSE LOCUS1* (*Ppd-H1*), is the central heading time gene regulated in responses to LD, at which recessive alleles (*ppd-H1*) reduce the response to LD [9]. Variation at *Ppd-H1* affects heading time of accessions originating from different geographical regions. Spring barley accessions originating from Middle East, e.g. tend to carry photoperiod responsive *Ppd-H1* alleles, causing early heading under LD, while the delay of heading time in Northern European accessions of spring barley is due to reduced photoperiod sensitivity, *ppd-H1*, alleles [1], [9]. Therefore, strength of photoperiod response is a key factor to understand the natural genetic variation and pathway of heading time in barley.

In addition to the *HvPRR*37 gene, which is located on the short arm of chromosome 2H [9], further genes of the heading time pathway have been identified in barley. *Ppd-H2*, responsive to short-day (SD) is located on 1HL, for which *HvFT3* has been proposed as a candidate gene [10]. Five *FLOWERING LOCUS T* (*FT*-like; *HvFT1-HvFT5*) genes were found in barley, and these genes play various roles during plant development through their photoperiod response, of which *HvFT1* has a major role in the transition from the vegetative-to-reproductive phase as an important source of variation in heading time [10]. Moreover, the *CONSTANS*-like (*CO*) gene family is known to regulate flowering time through the photoperiod pathway in *Arabidopsis* (a LD plant) and rice (SD plant). In barley, Griffiths et al. [11], and Cockram et al. [12] identified numerous homologs of *CO*-like genes (*HvCO1* to *HvCO18*) but their roles in the barley heading time pathway are still unclear. *GIGANTEA (GI)*-*CO*-*FT* is considered as a conserved central interaction partner in plant photoperiod pathway under LD, *Arabidopsis* [13]; however, the function of *HvGI* in the barley photoperiod pathway is still unclear. CCT domain gene families (CO, CO-LIKE, TIMING OF CAB1 (TOC1)), i.e. *CO*-like and *PRR*, have an important role in controlling heading time; in addition to these families [12] introduced uncharacterized genes carrying single CCT domains called *CCT MOTIF FAMILY* (*CMF*) genes. *PHYTOCHROME* (*HvPhy*) and *CIRCADIAN CLOCK ASSOCIATED* (*HvCCA*) genes clearly affect barley heading time pathway through interaction with other genes, such as *HvPhyC* which induces early heading by up-regulating *HvFT1* and bypassing *HvCO1* under LD [14]. In *Arabidopsis*, *LATE ELONGATED HYPOCOTYL* (*LHY*) and *CIRCADIAN CLOCK ASSOCIATED 1* (*CCA1*) suppress *FT* expression independent of the *CO* causing delayed flowering [15]. Similarly, *SHORT VEGETATIVE PHASE*-like (*SVP*-like) genes such as *VEGETATIVE TO REPRODUCTIVE TRANSITION gene2* (*HvVRT2*) in barley delayed heading time by inhibiting spike development under LD [16]. *RICE FLORICAULA/LEAFY* (i.e. *RFL*; syn. *ABERRANT PANICLE ORGANIZATION2*, *APO2*) is the homolog of *Arabidopsis LEAFY* and plays important roles in regulating the transition from vegetative to reproductive phase, maintenance of inflorescence meristem, floral organ identity/determinacy and flowering time in rice [13], [17], [18]. In *Arabidopsis*, *LFY* acts downstream of *SUPPRESSOR OF OVEREXPRESSION OF CONSTANS 1*, (*SOC1*) [13]; whereas *RFL* functions upstream of *OsSOC1* and reduced expression of *RFL* delayed flowering time in rice [18]. With regard to genes that are involved in responses to vernalization, *Vrn-H1* located on 5HL (promotes transition from the vegetative to the reproductive phase) is dominant in spring barley [19], while *Vrn-H2* (*HvZCCT*) located on 4HL delays heading in plants that have not been vernalized [20]; it similarly delays heading time under LD [21]. *Vrn-H3* (syn. *HvFT1*), a central integrator of different heading time pathways, had been identified on 7HS [22]. In addition, many independent *EARLINESS PER SE* (*EPS*) and *EARLY MATURITY* (*EAM*) loci have been identified in barley: *eps2S* (*eam6*) on 2HS, *eps3L* (*eam10*) on 3HL, *eps4L* on 4HL, *eps5L* on 5HL, *eps6L.1* and *eps6L.2* on 6HL, *eps7S* on 7HS and *eps7L* on 7HL [23]. The precise position of these genes in coherent barley heading time pathway is not yet understood.

High-throughput genotyping platforms recently developed in barley provide sufficient marker coverage to perform genome-wide association scans (GWAS) [24]. GWAS is a powerful tool for mapping complex plant traits, with unprecedented genetic resolution for gene identification in large-genome crops such as barley and wheat. GWAS can identify genes responsible for natural phenotypic variation through screening a large, diverse collection of accessions with high density genetic markers to find causal genes as a result of historical recombination [24]. In barley, GWAS has been used to identify single nucleotide polymorphism (SNP) markers associated with heading time [25], [26]. However, information on the genetic variation of pre-anthesis stages/sub-phases as key components of barley adaptation and grain yield is still lacking.

The aim of this study is to detect QTL underlying natural variation of pre-anthesis stages/sub-phases based upon differences in photoperiod response (*Ppd-H1*/*ppd-H1*) through dissecting time to heading into sub-phases in a world-wide spring barley collection. To achieve this objective, we phenotyped more than 3,000 plants at four developmental stages (AP, TIP, HD and AE) under controlled GH conditions, derived from 95 photoperiod responsive (*Ppd-H1*) and 123 accessions with reduced photoperiod sensitivity (i.e. *ppd-H1*), respectively. Distinction of these two photoperiod groups in our GWAS analysis allowed us to control population structure, while using a 9K SNP chip provided us with an unprecedented genetic resolution for studying the natural variation of time to heading. In combination with accurate phenotyping of pre-anthesis stages into sub-phases (i.e. sowing to AP, includes vegetative and early reproductive phases; AP-TIP; TIP-HD and HD-AE) within each photoperiod group, natural genetic variation of the time to heading could be genetically dissected resulting in the identification of novel QTL that were anchored to the barley physical map (e.g. several associations around *HvCO*-like genes). Clearly, novel rich genomic regions with highly associated SNPs were detected, which have not been detected before. This paper proposes a new heading-time model for barley with specific reference to allelic combinations to photoperiod-response groups.

## Materials and Methods

### The collection, genotyping and population structure

A collection of 218 world-wide spring barley accessions was used in this study. This collection includes 125 two- and 93 six-rowed accessions; i.e. 149 cultivars, 57 landraces and 18 breeding lines. The barley spike possesses three spikelets (one central and two lateral spikelets) per rachis internode. In six-rowed barley, all three spikelets are fertile, while the two lateral spikelets are sterile in two-rowed barley [27]. The origins of these accessions were from Europe (EU, 108), West Asia and North Africa (WANA, 45), East Asia (EA, 36) and Americas (AM, 29). This collection has been described by Haseneyer *et al.* [28] and more information is available under the following link: http://barley.ipk-gatersleben.de/ebdb.php3. In this study the collection was divided into two groups: *Ppd-H1* and *ppd-H1* accessions. These groups consist of two- and six-rowed cultivars, landraces and lines from different origins; more information about row-types and origins is provided in Table 1.

**Table 1:** Spike row-type and origins of spring barley accessions with photoperiod-sensitive (*Ppd-H1*) and reduced photoperiod sensitivity (*ppd-H1*).

Origin‡	Photoperiod-sensitive (*Ppd-H1*)	Six-rowed	Reduced photoperiod sensitivity (*ppd-H1*)				Total
	Two-rowed			Two-rowed		Six-rowed	
WANA	12	21		11		1	45
EU	10	6		80		12	108
EA	0	28		2		6	36
AM	6	12		4		7	29
Total	**28**	**67**		**97**		**26**	**218**
	**95**				**123**		

‡ WANA: West Asia and North Africa, EU: Europe, EA: East Asia, AM, Americas.

The collection was genotyped using the 9K iSelect SNP chip from Illumina, which was developed from RNA-seq data of 10 diverse barley cultivars [26]. Finally for our GWAS analyses, we focused on SNPs which had genetic and physical positions on the barley genome after quality control checking, filtering and evaluating 9K SNP [26], [29], [30]. In each group, only the SNPs with minor allele frequency (MAF) ≥5%were used for association analyses (4228 and 4050 SNPs for *Ppd-H1* and *ppd-H1* group, respectively). We used genetic marker positions anchored by physical map positions for SNP markers based on Barke x Morex RILs POPSEQ population [29].

**Figure 1:**
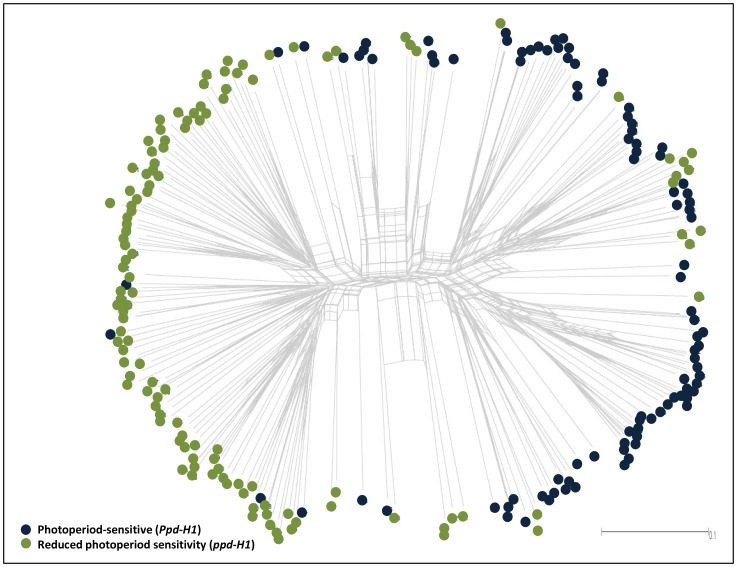
Population structure of 218 spring barley accessions based on 6355 SNPs information. 95 accessions showing photoperiod response (*Ppd-H1*) and 123 accessions with reduced photoperiod sensitivity (*ppd-H1*).

The population structure of this collection was determined by 6355 polymorphic SNPs (Figure 1). In this study, we divided this collection based on photoperiod response (*Ppd-H1* and *ppd-H1*) as major groups and geographical regions presented as sub-groups. Principal component analysis (PCA) was also used to infer the population structure in this collection. PCA is an indicator ordination tool for obtaining clusters, which can be explored visually in a two dimensional using GenStat 16 [31].

### Phenotyping

**Figure 2:**
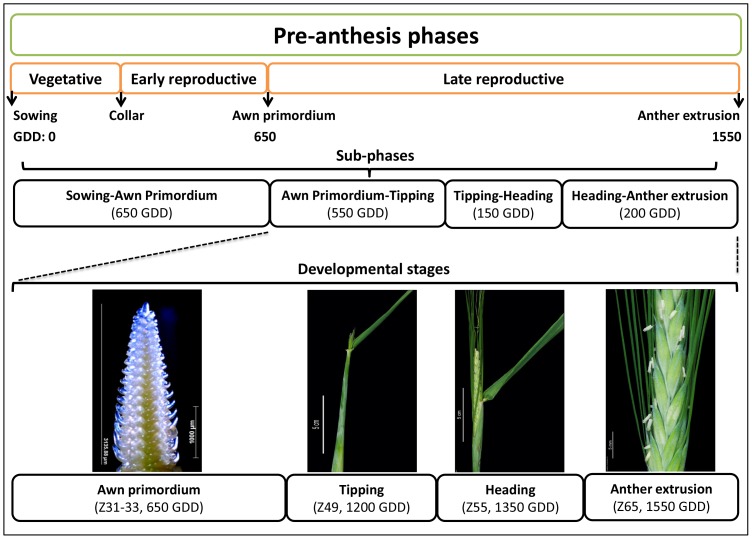
General figure of barley pre-anthesis phases. The figure includes the beginning developmental point from sowing to each stage (e.g. time to tipping is 1200 GDD; i.e. from sowing time to tipping stage) and the differences between stages (phase; e.g. tipping to heading phase (150 GDD); i.e. GDD for heading stage (1350 GDD) minus tipping stage (1200 GDD)). GDD is the average GDD in the whole collection (i.e. including both photoperiod groups). This figure also describes the developmental stages and sub-phases which form the late reproductive phase as described in (Alqudah and Schnurbusch 2014).

**Figure 3:**
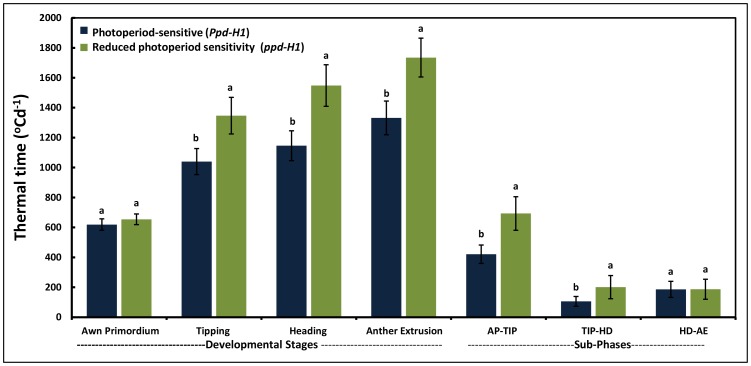
Thermal time for different developmental stages and sub-phases. Thermal time from sowing to the beginning of awn primordium (AP), tipping (TIP), heading (HD) and anther extrusion (AE) stages and thermal time of the duration of sub-phases. Letters differentiate between photoperiod-sensitive (*Ppd-H1*) and reduced photoperiod sensitivity (*ppd-H1*). The same letters are not significantly different at *P*≤0.05. Bars indicate standard deviation (n = 95 and 123 for *Ppd-H1* and *ppd-H1* barleys, respectively).

Thirty-five seeds of each accession from the collection of 218 spring barley accessions were germinated for 10 days under controlled condition in GH (LD condition, 16/8 h day/night and ∼20/∼16°C day/night). Seedlings were transferred to vernalization chamber (SD condition, 10/14 h and ∼4°C) for a period (28 days) when they reached 2–3 leaves stage. Afterwards seedlings were kept in an acclimation chamber for a period of 7 days (16/8 h and ∼14/∼12°C). Finally, strongest 30 seedlings of each accession were transplanted into 0.5 liter pots (one plant per pot; nine centimeter pot diameter and nine centimeter height) under GH condition. Plants were grown in a substrate containing peatmoss with 14: 16: 18/Nitrogen (N): Phosphorous (P): Potassium (K). To avoid any mineral deficiency each pot was additionally fertilized with 1.5 gram of solid fertilizer (that constitute minerals 17∶11∶10/N∶ P:∶K). Plants were irrigated daily and supplemental light (∼300 mE m^−2^s^−1^ PAR) was used to extend the natural light with low intensity incandescent lamps. Pots were randomized three times per week to reduce border and temperature-gradient effects on plant growth and development. The time for each stage was recorded when at least 50% of the main culm spikes in each accession had reached the stage: i.e. awn primordium stage, AP (Z31-33, maximum yield potential; Alqudah and Schnurbusch [3]); tipping stage, TIP (Z49, first awns visible on main culm); heading time stage, HD (Z55, half main culm spike emerged from flag leaf sheath); anther extrusion stage, AE (Z65, half of main culm spike with anthers; Zadoks et al. [32]); more information on individual stages can be obtained in Figure 2 and [3]. To identify the exact timing for the AP stage, regular microscopic dissection and image capture (three times per week) from immature barley main culm spikes was performed by using Stereo Microscope Stemi 2000-C with KL 1500 LCD; Axio Vision, 4.8.2, ZEISS, Germany.

Thermal time (°C.D^−1^) or growing degree-days (GDD, base temperature was 0°C) from sowing to reach each stage was recorded to measure the required days/thermal temperature for each stage and the duration between the stages (Table S1). The phenotypic analyses of pre-anthesis sub-phases (i.e. dissection work) had to be performed in eight batches due to limited GH space and feasibility of workload. Each batch contained thirty accessions plus two check lines (Morex and Barke); hence, all 218 accessions were grown from September 2011 to April 2012 in eight consecutive batches for dissection and phenotypic, stage-specific data collection under controlled GH conditions. Each batch of accessions had a completely randomized design with 30 plants per accession. At least three biological replications at each developmental stage were recorded for analysis. The collected data were analyzed using SAS software version 9.3 at probability level *P*≤0.05. *Student's t-test* was used to compare between *Ppd-H1* and *ppd-H1* groups. REML (Residual Maximum Likelihood) was used to analyze phenotypic data by SAS software [33]. Best Linear Unbiased Estimates (BLUEs) were used to estimate phenotypic means for each trait in individual accession and estimated means were used for association analysis. Broad-sense heritability for traits in each group was calculated across growing times as the ratio between the genetic variance and the phenotypic variance which includes genotypic by growing times (environments) interaction and error variance components using PROC VARCOMP [33].

### Genome-wide association study (GWAS) analysis

GWAS of two groups: 95 *Ppd-H1* and 123 *ppd-H1* accessions were identified using their corresponding genotype datasets according to the G/T SNP22 [9]. The association of phenotypic traits (BLUEs) and each single marker was analyzed by mixed linear model (MLM) implemented using GenStat 16 [31]. Eigenanalysis with Single Trait Association Analysis (Single Environment) was used as correction for population structure in MLM for accounting relatedness to avoid false positives in GWAS as described by Tondelli et al. [34]and Pritchard et al., [35]. A threshold of *P-value* 0.01 was used in all traits detecting significant SNPs with -log_10_ *P-values* ≥2. Such a set of significant SNPs (i.e. exceeding the -log_10_ *P-values* ≥2) underwent another test of robustness using the false discovery rate (FDR) at 0.05 [36]. FDR analysis provided highly significant associations (*P-values* ≥ FDR) among which significant *P-values* from ≥2 and lower ≤ FDR; this technique has been widely applied to GWAS analyses; see review by van den Oord [37]. Allele effects were estimated relative to the performance of “Mansholt zweizeilig” and “Isaria” cultivars for *Ppd-H1* and *ppd-H1* groups, respectively [34]. To validate our association results, we analyzed all stages under GH condition for comparing the significant loci with association from the same collection under field conditions [25], [30]. Genetic maps were drawn using MapChart 2.2 Windows [38] using those SNP markers passing the FDR threshold to determine highly associated QTL within confidence interval ±5 cM. Known heading time genes (bold and italicized) have been genetically anchored and located according to the Barke x Morex RILs (POPSEQ) sequence contigs; more information about these genes, their accession numbers and genetic chromosome positions are available in Table S2.

## Results

### Population structure of a world-wide spring barley collection

The population structure of this collection was determined using polymorphic SNP data from the 9K array. To this end, we divided the collection into two groups based on the presence of a single diagnostic SNP in *HvPRR37*, thereby separating photoperiod responsive (i.e. photoperiod-sensitive, *Ppd-H1*) accessions from those with reduced photoperiod sensitivity (i.e. *ppd-H1*) [9]; Sharma *et al. in preparation*; Figure 1). The relationships among spring barley accessions were inferred using principle component analysis (PCA). The genetic variation between groups was explained by PCA and the collection was clearly separated into *Ppd-H1* and *ppd-H1* spring barleys based on heading time data (Figure S1). PCA-1 explained 28.1% of variation and separated the *ppd-H1*-group from the *Ppd-H1-*group, with few exceptions, clearly showing greater genetic variation among accessions from the *Ppd-H1*-group compared to *ppd-H1*-carrying accessions (Figure S1). Interestingly, the genetic variation at heading time could be further subdivided based on geographic origins (Figure S2). The European (EU) accessions clustered from the remaining regions with few exceptions (Figure S2). Although a significant proportion of the collection clustered separately based on photoperiod response, row-type classes formed another determinant within the geographic and photoperiod groups. Notably, most of the accessions in the *Ppd-H1-*group are six-rowed barleys from West Asia and North Africa (WANA) and East Asia (EA), while most of the accessions in the *ppd-H1-*group are two-rowed barleys from EU (Table 1). Generally, these results suggest that the spring barley collection (218 accessions) is separable into two major groups based on the response to photoperiod and reduced photoperiod sensitivity (*Ppd-H1*/*ppd-H1*) at heading time.

### Natural phenotypic variation in pre-anthesis developmental stages and sub-phases

It is difficult to understand the full complexity of the time to heading in cereals by only studying the period from sowing until heading/flowering. Our analyses aimed at examining associations for particular pre-anthesis stages or sub-phases, to maximize the likelihood of finding new associations. Hence, we developmentally dissected the pre-anthesis time of barley into four stages (AP, TIP, HD and AE) and four sub-phases (sowing-AP, AP-TIP, TIP-HD and HD-AE; Figure 2). The first investigated pre-anthesis stage was AP. Plants at this stage had already passed the vegetative-to-reproductive transition and finished early spike differentiation. The time from sowing to AP represents approximately 40% of the entire time to AE in barley and on average it took 650 growing degree-days (GDD, Figure 2). Late reproductive development in barley can be further sub-divided into three sub-phases, of which AP-TIP is the longest phase with an average of 550 GDD (Figure 2).

**Figure 4:**
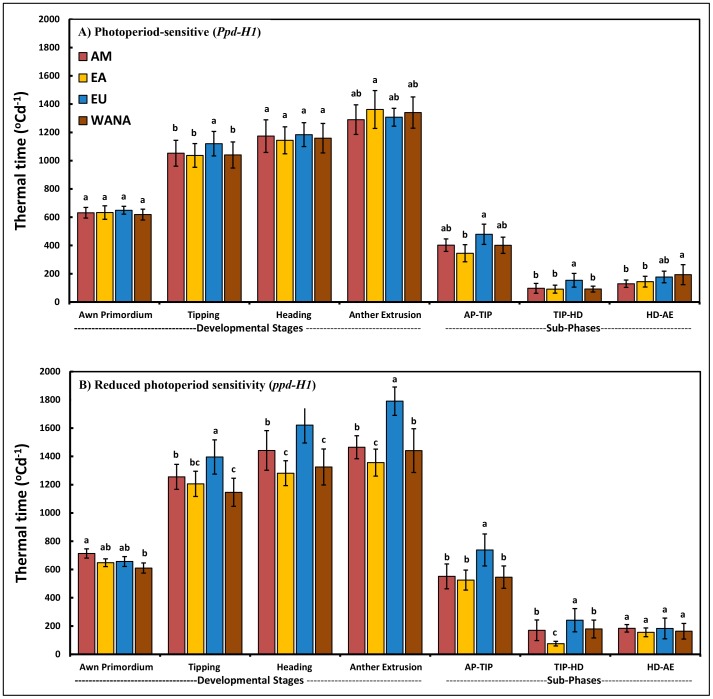
Thermal time for different developmental stages and sub-phases. Thermal time from sowing to the beginning of awn primordium (AP), tipping (TIP), heading (HD) and anther extrusion (AE) stages and thermal time of the duration of sub-phases. Letters differentiate between origins within photoperiod-sensitive (*Ppd-H1*) and reduced photoperiod sensitivity (*ppd-H1*). The same letters are not significantly different at *P*≤0.05. Bars indicate standard deviation. A). Number of *Ppd-H1*-carrying accessions for WANA  = 33, EU  = 16, EA  = 28 and AM  = 18. B). Number of accessions with reduced photoperiod sensitivity (*ppd-H1*) for WANA  = 12, EU  = 92, EA  = 8 and AM  = 11.

Comparisons of thermal time to reach different developmental stages in *Ppd-H1* and *ppd-H1* spring barley groups under GH condition yielded highly significant variation between the groups (*P*≤0.05; Figure 3 and Table S1). The duration from sowing to reach each developmental stage was significantly longer in the *ppd-H1*group at all stages except AP stage; i.e. at TIP, HD and AE the differences were +302, +406 and +373 GDD, respectively, in favor of the *ppd-H1* group (Figure 3). Separate analysis of thermal time to reach developmental stages between geographical regions in *Ppd-H1* and *ppd-H1* spring barley groups can also explain the genetic variation (Figure 4 A and B). In the *Ppd-H1* group, there was no clear trend of results between geographical regions (Figure 4A), whilst the genetic variation between geographical regions were much clearer in the *ppd-H1* group (Figure 4B). In particular EU accessions of the *ppd-H1* group had the longest durations from sowing to different pre-anthesis stages (TIP, HD and AE stages) except AP stage (Figure 4B).

Durations between sub-phases can explain the significant variation between the two photoperiod groups. The duration between AP-TIP was the longest late-reproductive sub-phase in both groups resulting in 690 GDD for the *ppd-H1*-carrying accessions but only 400 GDD for the *Ppd-H1* group (Figure 3). The duration between TIP-HD and HD-AE was not significantly different between both groups. Generally, the AP-TIP sub-phase is the most important developmental period related to the observed genetic variation for the time to heading between these groups. In terms of origin, EU accessions had the longest duration between AP-TIP and TIP-HD sub-phases in both groups (Figure 4A and B). The genetic variation in pre-anthesis stages between geographical regions became clearer in the *ppd-H1* group. Thus, the results of our dissection of the pre-anthesis stages provide a promising route to explain the genetic variation between the photoperiod groups (*Ppd-H1* and *ppd-H1*) in barley.

Notably, the broad-sense heritability values for pre-anthesis developmental stages and sub-phases in each photoperiod group (*Ppd-H1* and *ppd-H1*) were above 0.88, indicating that the traits related to pre-anthesis phase duration are highly heritable (Table 2). Due to the very high heritability values associated with pre-anthesis stages, we are able to detect particular QTL for each stage and sub-phase within each photoperiod group.

**Table 2:** Estimation of broad-sense heritability (*H^2^*) for developmental stages and sub-phases measured as thermal time ^○^C.D^-1^ (GDD) in the association mapping groups.

Stage/phase†	Photoperiod-sensitive (*Ppd-H1*)	Reduced photoperiod sensitivity (*ppd-H1*)
**Awn primordium (AP)**	0.92	0.90
**Tipping (TIP)**	0.91	0.92
**Heading (HD)**	0.90	0.92
**Anther extrusion (AE)**	0.91	0.93
**AP-TIP**	0.89	0.91
**TIP-HD**	0.92	0.90
**HD-AE**	0.90	0.88

† AP: awn primordium [3]; TIP: tipping, Z49; HD: heading, Z55; AE: anther extrusion, Z65 [32].

*H^2^*: broad-sense heritability for each group overall growing times based on accessions mean.

n = 95 and 123 for barleys with photoperiod-sensitive and reduced photoperiod sensitivity, respectively.

Developmental stages calculated based on thermal time °C.D^−1^ (GDD).

### Identification of natural genetic variation for pre-anthesis development using GWAS - Strategies for validating and improving GWAS analyses

We performed GWAS for all developmental stages under GH conditions on the collection to compare it with previous results of the same collection obtained from multiple field evaluations [25], [30]. In our study, the major locus for HD (*Ppd-H1*) appeared to be identical to the previously known locus, for instance at HD stage (Figure S3), clearly indicating that field and GH data are comparable. Compared to the previous study [25], the power of our GWAS to detect associated loci was increased, likely as a result of more controlled growing conditions (e.g. GH, higher heritability) and the higher number of SNP markers (9K). These results re-confirm that GH conditions are appropriate for studying pre-anthesis phase durations [3].

**Figure 5:**
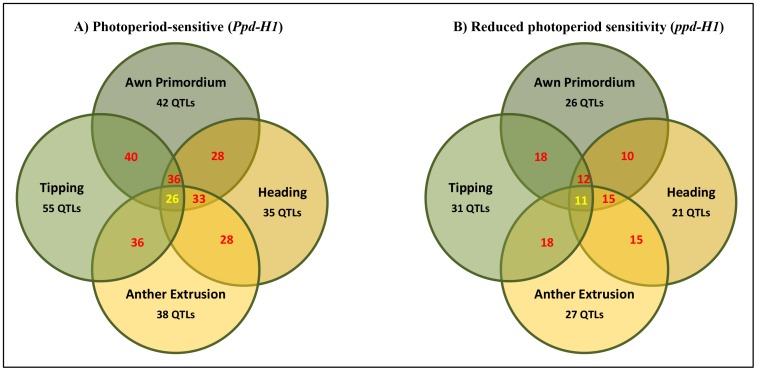
Number of QTLs (within confidence interval ±5 cM) overlapping between developmental stages in groups carrying A) photoperiod-sensitive (*Ppd-H1*) and B) reduced photoperiod sensitivity (*ppd-H1*). Number in red denotes the number of shared QTLs between each pair of developmental stages. Number in yellow denotes the number of shared QTLs between all developmental stages. QTLs exceeding significance level (-log_10_ (*P-value*  = 0.01)) are considered as significantly associated.

GWAS was conducted for each photoperiod group (*Ppd-H1* and *ppd-H1*) independently using SNPs derived from the 9K array (Figure S4 and S5). Association analyses between each SNP and thermal time between developmental stages/sub-phase were performed using mixed models to generate Manhattan plots (Figure S4 and S5). Although the number of significant SNPs were higher in the *Ppd-H1* group (i.e. SNP marker, -log_10_ >2, *P*-value 0.01; Figure S6), the association signals and map resolution were much clearer in the *ppd-H1* group (123 accessions) than in the *Ppd-H1* group (95 accessions) likely as a result of accession number. Because of the high number of shared QTL (defined as confidence interval ±5 cM) between developmental stages (Figure 5) and sub-phases (Table S3), we reduced the number of spurious associations by only considering those SNPs, which exceeded the false discovery rate (FDR; Figure S7). To increase the precision-power of our GWAS analysis (genetic SNP positions), we used the latest version of the barley physical map [29]. This map provided great power to locate physical/genetic positions of known heading time gene(s) and/or QTL(s) for each developmental stage/sub-phase.

### Identification of marker-trait association within the photoperiod-sensitive (*Ppd-H1*) group

**Figure 6:**
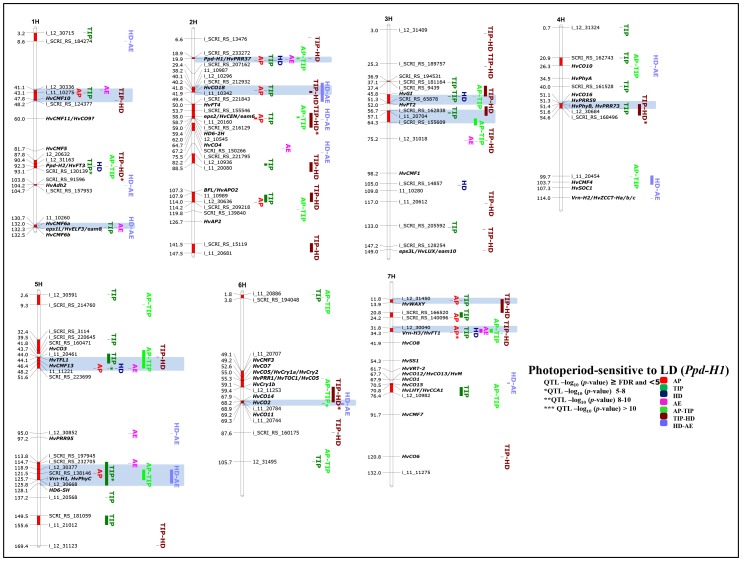
Genetically anchored position of highly associated QTLs at all barley developmental stages and sub-phases in the photoperiod-sensitive (*Ppd-H1*) group using 9K SNP markers. Bold and italicized gene names indicate genetically anchored positions of known heading time genes in the Barke x Morex RILs. Associated chromosomal regions are highlighted with different colors according to stages and sub-phases. Red chromosomal areas indicate the range of significantly associated QTLs (within confidence interval ±5 cM) which are exceeding FDR level of each developmental stage or sub-phase. Highlighted chromosomal regions in light blue denote group-specific associations.

All 95 accessions in this group possess functional *Ppd-H1* alleles and so display a strong response to long day (LD) condition. GWAS analysis in this group detected in total thirty significantly associated chromosomal regions, of which twelve are group-specific and only occur here, indicating that *Ppd-H1*-carrying accessions exhibit a complex genetic architecture for pre-anthesis development. Another specific feature of this group is the high number of significant associations for the time to AP (> FDR for AP, i.e. ten regions on four chromosomes; Figure 6), suggesting that there is ample natural genetic variation in the duration for this first sub-phase among accessions. Many of these chromosomal regions very precisely co-localized with known heading time genes (e.g. 7HS 31.8-34-3, *Vrn-H3/HvFT1* having the most significant effect until AP; or 2HS 26.8–31.0 cM, *Ppd-H1*/*HvPRR37*; Figure 6) and novel candidate gene regions (i.e. 1HS 41.1–48.2 cM, incl. *HcCMF10*; 2HS 38.2–41.9 cM, *HvCO18*; 5HS 43.7–51.6 cM, *HvCO3*, *HvTFL1* (barley *TERMINAL FLOWER 1*), *HvCMF13*; 7HS 11.8–13.9). Many *CO*-like genes are among the chromosomal regions controlling early development until AP, suggesting that these family members promote the vegetative-to-reproductive phase transition and early spike development. All of these *CO*-like genes also showed highly significant associations with later developmental stages and sub-phases (i.e. TIP, HD, AP-TIP etc.) except for the two chromosomal regions on 4H (51.1–54.6 cM, *HvCO16*, *HvPRR59*, *HvPhyB*, *HvPRR73*; Figure 6) and 6HL (67.9–69.3 cM, *HvCO14*, *HvCO2*, *HvCO11*), which were only detectable during later developmental stages/sub-phases in this group. Other interesting associations were found in the centromeric region of 2H (*HvFT4*, *HvCEN*, *HvCO4*, *HD6-2H*), for the barley ortholog of *RICE FLORICAULA/LEAFY* (*BFL*) on 2HL (107.3 cM, *BFL*; Figure 6) and on chromosome 5HL (*Vrn-H1*, *HvPhyC*) all affecting early (AP and TIP) and later development in this group. For one region on 7HS (20.8–24.2 cM) showing significant associations with time to AP and TIP, we were not able to locate known flowering time genes.

It was previously shown that the duration between AP-TIP is positively related with single-plant yield and yield components in barley [3]. For this second sub-phase (i.e. AP-TIP), we found in total eighteen out of thirty significant associations (i.e. ∼60% from the total identified QTL) within the photoperiod-sensitive (*Ppd-H1*) group (Figure 6), suggesting the existence of abundant genetic variation for breeding. Among these regions, the highly associated SNPs around *HvCO14*, *HvCO2* and *HvCO11* (6HL, 67.9–69.3 cM; Figure 6) showed the strongest phenotypic effect on the time from AP to TIP (-80 GDD) and occurred exclusively in this group. GWAS analysis of the time to TIP identified thirty significant associations (Figure 6). The time to TIP can be seen as an integrator of the two sub-phases sowing-AP and AP-TIP. Hence, it is not surprising that many associations overlap with AP, AP-TIP and later stages and sub-phases, indicating that many of these loci affect more than one stage or sub-phase. Nevertheless, we found three chromosomal regions in this group, which had major phenotypic effects on the time to TIP: these were highly associated SNPs around the *Ppd-H2* locus on 1HL (87.8–93.1 cM; Figure 6), and two regions on chromosome 5H, containing *HvCO3*, *HvTFL1* and *HvCMF13* (5HS, 43.7–51.6 cM) and the *Vrn-H1, HvPhyC* (113.8–125.8 cM). The two late sub-phases, i.e. TIP-HD and HD-AE, are relatively short compared with the other two earlier sub-phases (Figure 3); but we were still able to find twenty-five and thirteen significantly associated chromosomal regions, respectively (Figure 6). As expected, the majority of these loci preferentially co-located with regions being associated with other developmental stages and sub-phases, again reinforcing the fact that a high degree of pleiotropic gene actions exists between several stages and sub-phases.

### Identification of marker-trait association within the *ppd-H1*-carrying group

**Figure 7:**
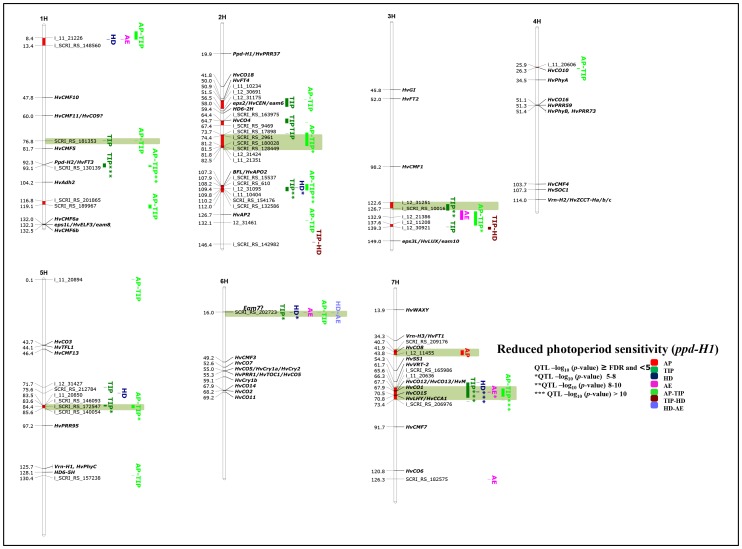
Genetically anchored position of highly associated QTLs at all barley developmental stages and sub-phases in the group carrying reduced photoperiod sensitivity (*ppd-H1*) using 9K SNP markers. Bold and italicized gene names indicate genetically anchored positions of known heading time genes in the Barke x Morex RILs. Associated chromosomal regions are highlighted with different colors according to the stages and sub-phases. Red chromosomal areas indicate the range of significantly associated QTLs (within confidence interval ±5 cM) which are exceeding FDR level of each developmental stage or sub-phase. Highlighted chromosomal regions in light green denote group-specific associations.

All 123 accessions in this group carry the *ppd-H1* allele with reduced photoperiod sensitivity and thus reach most stages/sub-phases significantly later under LD (Figure 3). GWAS analysis in this group identified in total at least twenty significantly associated chromosomal regions of which only seven were group-specific, indicating that *ppd-H1*-accessions display a much less complex genetic architecture for pre-anthesis development (Figure 7). Among those regions, most highly associated SNPs around *HvCO1* (7HS, 67.6–73.4 cM; Figure 7) resulted in the major phenotypic effect in this group. However, *HvCO1* resides in a chromosomal region, which physically contains three other *CO*-like family members (*HvCO12*, *HvCO13/HvM* and *HvCO15*) and the circadian clock-related genes, *HvLHY*/*HvCCA1*. Nevertheless, specific associations could be found for several physically anchored and co-located SNPs for each of the genes therefore making it possible to attribute phenotypic effects to individual genes. Hence, the most significant effects were found for SNPs co-locating with *HvCO1* for the time between AP-TIP***, time to TIP***, HD*** and AE* (Figure 7) thereby shortening duration, i.e. promoting heading, by −126, −148, −172 and −153 GDD, respectively. The physically close *HvLHY*, *HvCCA1* region, however, showed up in both groups (Figure 6 and 7) a consistent heading time-inhibiting effect (i.e. here in the *ppd-H1* group at TIP +128 and +105 GDD for *HvLHY*, *HvCCA1*, respectively), suggesting that these two tightly linked chromosomal regions on 7HS function reciprocally on *HvFT1* expression. Another very consistent but group-specific effect was obtained from SNPs on 6HS (16.0 cM; Figure 7), reducing time to TIP* and HD* by −151 and −173 GDD, respectively. For the last two specific regions in this group with highly significant associations we did not succeed to locate any known candidate genes (3HL, 122.6–126.7 cM; 5HL, 83.5–85.6 cM). All of these four group-specific regions have in common that they primarily affect later developmental stages and/or sub-phases (Figure 7). Importantly, in this group there was only one region associated with SNPs around *HvFT1* on 7HS (34.3–43.8 cM; Figure 7), which showed significant associations with time to AP (i.e. first sub-phase). All other detected regions affected later developmental stages or sub-phases (e.g. thirteen for AP-TIP, ten for TIP, three for TIP-HD, six for HD; Figure 7), suggesting that genetic variation for later developmental stages/sub-phases is more relevant in *ppd-H1*-carrying accessions.

## Discussion

The new approach of combining phenotypic dissection of pre-anthesis development with a high-density marker scan provided an unprecedented opportunity to better understand the genetic basis of time to heading in barley, representing a cereal crop species of worldwide importance. Splitting the mapping population based on photoperiod response (*Ppd-H1* and *ppd-H1*) for GWAS analysis revealed a comprehensive network of QTL that can be directly used to refine heading time pathways. Following this approach we were able to detect novel stage- and sub-phase-specific associations, which otherwise would not have been found by simply scoring heading time.

### A refined strategy for studying time to heading

It is essential to clearly define pre-anthesis phases and explain the importance of this refined approach for future studies. Previous works defined pre-heading phases in barley as leaf/spikelet initiation phase (from sowing to the onset of jointing) and stem elongation phase [6], [7], which most likely lead to an overlap between vegetative and reproductive phases. Alqudah and Schnurbusch [3] proposed a new approach for studying pre-anthesis spike development, which enables a more precise detection of specific stages and sub-phases. This approach appears advantageous because it precisely defines pre-anthesis phases to developmental intervals, and hence allows the study of genetic factors controlling pre-anthesis stage and sub-phase durations. We anticipate that follow-up work for studying time to heading in cereals will gain value-added information while following this dissection-approach.

### The effectiveness of SNP array, population structure and GWAS

**Figure 8:**
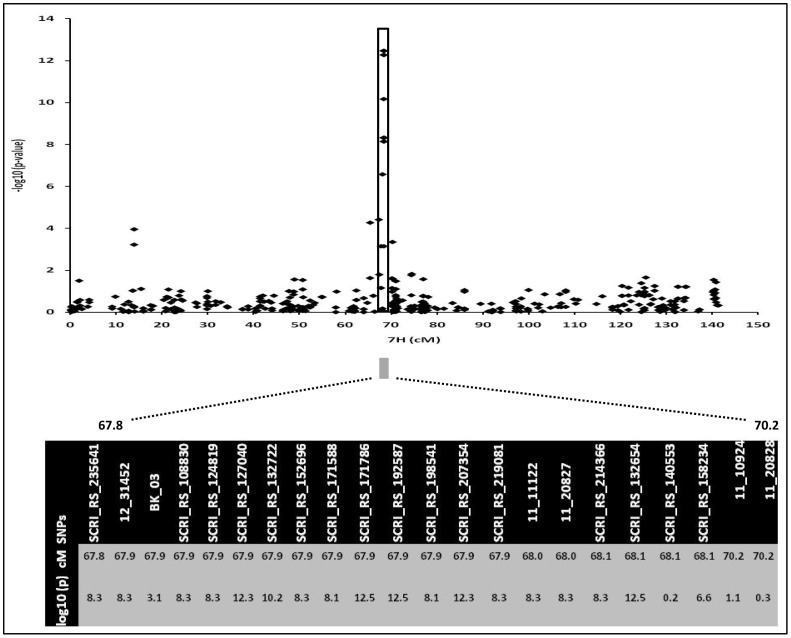
Regions of chromosome 7H showing association signals of the candidate gene (*HvCO1*) at the heading stage. The top of the panel shows the region of the SNP marker peak (-log_10_ (*p-value*)). The lower panel zooms into a narrow region for the candidate region with the position and -log_10_ (*p-value*) of highly associated markers which are co-located with *HvCO1* (67.9 cM).

Obtained association signals and the power of GWAS were much more informative when the allelic status at *Ppd-H1* was considered for population structure. The results show that SNP array density (9K) and collection size were sufficient to identify highly significant marker-trait associations. We compared previously identified heading time-related associations/QTL using the present spring barley collection studied with 957 SNPs [30] and results from bi-parental populations [6], [7]. For heading time, we similarly detected all previously identified QTLs when using the larger SNP array (9K) but most importantly several new QTL underlying pre-anthesis spike development. Compared to previous studies, the power of our GWAS to detect associated loci was increased, likely as a result of better genome coverage and more SNPs. The power of GWAS can be strongly attributed to the *Ppd-H1*-based population structure, which had not been applied before to study natural variation in heading time for barley. This approach provides strong associations, especially when *Ppd-H1* alleles were less active thereby revealing the importance of *HvCO1* as potential candidate gene. Notably, among the 13 most highly associated SNP markers for this locus one *HvCO1*-specific marker (BK_03; Figure 8 and Table S2) was detectable, clearly indicating that our GWAS analysis reached a high predictive capability. Moreover, specific gene-derived marker-trait associations were found in two other cases, thereby validating effects seen at *Vrn-H3/HvFT1* and *Ppd-H1* (Table S2).

In this study, high broad-sense heritability (Table 2) and many shared QTL between developmental stages (Figure 5 and Table S3) confirmed that these stages are genetically and pleiotropically controlled. This is most likely the result of precision-phenotyping under stable GH conditions, using pre-anthesis stages and sub-phases, natural population structure (differences in photoperiod response) and larger number of SNP markers, including their precise physical location. A combination of all of these findings empowered us to reveal new QTLs/genes for natural variation in the time to heading for barley.

### Heading time genetic network models

**Figure 9:**
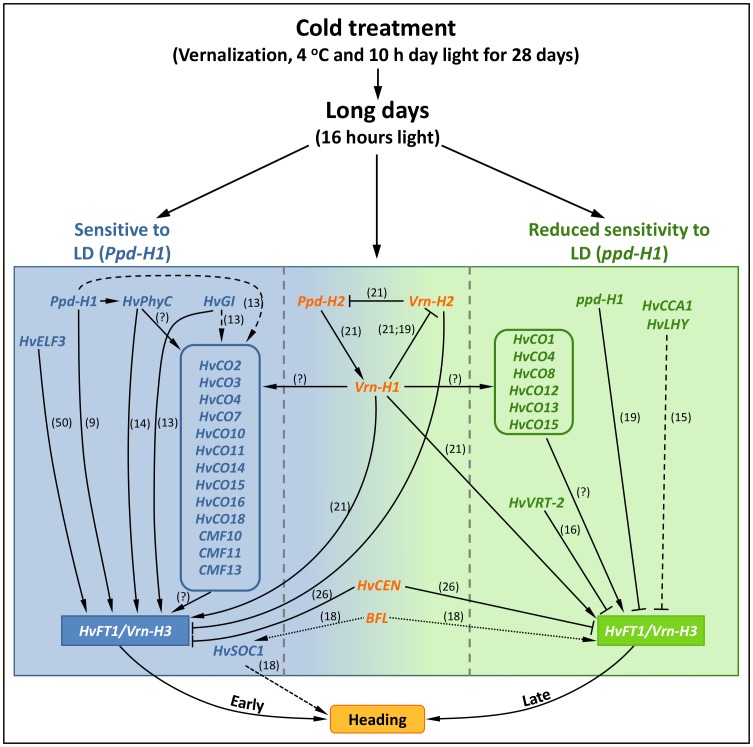
Model of heading-time regulation in both photoperiod groups (*Ppd-H1*; *ppd-H1*) under long day (LD) condition. Arrow heads indicate promotion of heading; whereas flat arrow heads indicate delay of heading. Genes with known roles in the regulation of heading time in barley are shown by continuous lines. Known interaction from Arabidopsis is shown in dashed lines. Known interaction from rice is shown in round dotted lines. Ambiguous interaction is indicated by a question mark. Numbers in parenthesis show the reference to published interaction.

Our GWAS analysis revealed several genetically anchored genes being involved in the regulation of heading time. Based upon the detected candidate regions in combination with previous knowledge on these genes we developed genetic network models for the two photoperiod groups (Figure 9), including genes which play a role in both groups (center panel of Figure 9).

Previous studies clearly established that *Ppd-H2* induces *Vrn-H1* under LD conditions, which in turn up-regulates *HvFT1* expression thus promoting heading ([21]; Figure 9). Natural variation around these three loci consistently showed phenotypic effects in our world-wide spring barley collection regardless of photoperiod status but with *Ppd-H2* as the most important locus. Almost all developmental stages and sub-phases were significantly associated with the *Ppd-H2* chromosomal region within the *Ppd-H1* group, clearly indicating that this locus is important in photoperiod-respons and vernalized spring barleys under LD. It was shown that the earliness effect of *Ppd-H2* is repressed through *Vrn-H2* expression under LD [21]. However, we were not able to find significant associations at *Vrn-H2*, suggesting that this locus is either deleted, or present at very low frequency in our collection. Furthermore, significant associations in the *ppd-H1* group for TIP and AP-TIP fits well to the notion that *Ppd-H2* primarily affects early developmental stages or phase transition [39] and has less relevance during later stages.

The spring allele at *Vrn-H1* is known to inhibit *Vrn-H2* and was associated with earlier heading [19], [21] which is consistent with *Vrn-H1* promoter-GFP expression analysis [40]. Interestingly, we used vernalized spring barley but still observed significant associations at *VrnH1* in the *Ppd-H1* group, which may suggest rich allelic variation at *Vrn-H1* or/and *HvPhyC* in this group. It is known that *Vrn-H1* expression can vary due to deletions within its first intron [41]-[44], suggesting that quantitative differences in phase duration could be related to differences in *Vrn-H1* transcript levels; however, further work is still required to ascertain the allelic effects of the *Vrn-H1, HvPhyC* locus. No allelic variation within the *ppd-H1*-carrying group occurs at *Vrn-H1*, *HvPhyC* rather suggesting selection for late-heading time alleles within this group. Whether *Vrn-H1* also promotes heading through induction of *CO*-like gene family members is yet unknown (Figure 9; arrow with “?”). Allelic variation around *BFL* appears as the first report for temperate cereals that this important phase-transition-gene is relevant in both photoperiod groups. Postulating a similar role for *BFL* in rice [18], heading time control is very likely promoted particularly in the earlier heading and *Ppd-H1* group through a combination of allelic variation found at *HvSOC1* ([18], Figure 9). However, we did not find any associations for *HvSOC1* within the *ppd-H1* group; this might be explainable through the fixation of only one allele-type. In rice, higher transcript levels of *RFL* had been causal for early flowering [18]. Later heading time of the *ppd-H1* accessions suggests that *BFL* transcript levels may be lowered in this group, thereby resulting in an extended AP-TIP period. Thus, *BFL* appears to be an important gene affecting late sub-phase duration, especially in *ppd-H1*. Significant SNPs around *HvCEN* also became detectable in both groups, indicating that the heading time delaying effect of this gene (*Ppd-H1*: +60 GDD; *ppd-H1*: +138 GDD) has been manifested in our spring barely collection. However, Comadran et al. [26] reported that natural protein variation at *HvCEN* is low and only revealed two functional haplotypes in cultivated barleys (i.e. p.Pro153Ala substitution). Assuming a very similar haplotype structure at *HvCEN* in our spring barley collection may be sufficient to explain obtained results. However, we cannot completely rule out the possibility that the observed effect has been derived from another physically linked unknown gene, which is in linkage disequilibrium with *HvCEN* in the large centromeric region of chromosome 2H [26].

Barley plants carrying *Ppd-H1* alleles respond to LD conditions through up-regulation of *HvFT1* resulting in early heading [9]. For this group we found significant association around the *Ppd-H1* locus (i.e. the *HvPRR37* gene) affecting all stages and sub-phases. This finding can mainly be explained through the presence of natural variants around *Ppd-H1* in our collection (Sharma et al. *in preparation*). Similarly, natural variation at *HvFT1* consistently accounted for heading time effects in this group, further corroborating previous findings that natural copy number variation at *HvFT1* contributes towards accelerated heading time in barley [45]. Overall, we found that the *Ppd-H1* group exhibited a complex genetic constitution for pre-anthesis spike development and final time to heading. One reason for the high degree of complexity in this group is the involvement of 10 genes belonging to the *CO*-like family, which co-located with at least one significant association in our analysis (Figure 9). *CO*-like genes usually regulate heading time by photoperiod in barley [11], [12]. However, in the past it was not possible to specifically assign *CO*-family members to particular photoperiod groups, or to estimate their possible role in a stage or sub-phase dependent manner [1]. Following our strategy of genetically dissecting pre-anthesis development it becomes possible to specifically relate allelic variation around *CO*-like family members to specific photoperiod groups and developmental stages (Figure 9). One important example among *Ppd-H1* accessions is the *CO*-like gene cluster on 6H (*HvCO2*, *CO11*, *CO14*; 67.9–69.3 cM) which mainly affects phase durations after AP stage. More detailed SNP analyses suggest that the effect is more closely related to the *HvCO2*-*HvCO11* region; however, future functional work on individual genes is required to show whether one or more genes in this cluster contribute to variation in pre-anthesis development and time to heading. Similar conclusions can be drawn from gene clusters on chromosome 4H, which besides *HvCO16* also includes *HvPRR59*, *HvPhyB* and *HvPRR73* (see 4H: 51.1–51.4 cM), 5HS (including *HvCO3*, *HvTFL1*, *HvCMF13*; 43.7–51.6 cM) and 1HS (*HvCMF10*; 41.1–48.2 cM). Most interestingly, associations found for all of these regions exclusively occurred in the *Ppd-H1* group, indicating that these chromosomal regions harboring *CO*-like and *CMF* genes may play a major role in adaption for early heading time in barley. Another group-specific effect was found for SNPs around the *FT*-like homolog *HvFT2* and the circadian clock-related gene *HvGI* (3HS: 45.8–52.0 cM) both promoting time to heading. *HvGI* was detected with minor effect, supporting the notion by Dunford et al. [46] that *HvGI* does not provide a major source of adaptive variation in photoperiod response. Nevertheless, we assume that *HvGI* may have an important role in this group by promoting pre-anthesis spike development through the conserved *GI-CO-FT* pathway [13].

Taken together, these results suggest a central role of *HvCO-like* genes in the early heading time pathway under LD in photoperiod responsive spring barley accessions. We noted that some of the associations identified in this group are of minor effect with *P*-values just passing significant thresholds, which are more likely affected by variation in response to LD, gene interaction and/or geographical origin effect. Compared to previous studies, controlled GH conditions in conjunction with the novel pre-anthesis dissection approach and high-resolution physical map information draws a much more refined picture of natural adaption to photoperiod in barley.

Less active alleles of *HvPRR37* (i.e. *ppd-H1*) delay heading time under LD by reduced apex and spike development [19], at which *ppd-H1* alleles mainly alter expression levels of *HvCO1* as a result of changed circadian timing [9]. In this study, the highly significant effect of *HvCO1* on pre-anthesis spike development and time to heading was markedly associated only within the *ppd-H1* group. Although its genetic position strongly suggests that the strongest associations reside very close to *HvCO1* (supported by 13 SNP markers; see Figure 9), we cannot completely rule out that linkage with other *CO* genes in this region like *HvCO12, HvCO13/HvM* and *HvCO15* (Figure 7) contribute to this effect. Natural variation around this gene possibly provides the genetic basis for a large portion of the observed differences in pre-anthesis development within this late-heading group (*ppd-H1*). Following conclusions by Turner et al. [9], it can be anticipated that early-heading in this group is most likely coupled with higher *HvCO1* transcript abundance, thus promoting heading. To verify this hypothesis future work is needed to test structural and functional differences at this locus. Among other highly significant group-specific regions the association for promoting heading on 6HS (16.0 cM) stood out. Previous work identified that the *eam7* mutant phenotype had been linked to a region on 6HS, now establishing this position as a candidate for this mutant [47]. Moreover, we found three important chromosomal regions on 2H (107.3–112.0 cM), 3HL (122.6–126.7 cM) and 5HL (83.5–85.6 cM), which showed group-specific associations but did not co-locate with known candidate genes. The 3HL region is approximately. 30 cM proximal to the known circadian clock-related *HvLUX* gene [48]. More future work is required to validate this association.

The number of fewer but more significant associations identified in this group (i.e. *ppd-H1*) could be due to the fixation of heading time alleles, or due to the larger number of accessions used in this group (123) and/or longer duration of developmental stages. Only highly significant association around *Ppd-H2*, *HvCO1* and *BFL* genes promote heading within this group. This suggests that within this group the genetic architecture of heading time is less complex and that three genes play a major role on heading time compared to several genes in the *Ppd-H1* group. Another argument could be that within the *ppd-H1*-group *Ppd-H2* might have taken over the effect of *PpdH-1*, and *HvCO1* substitutes the *CO*-family gene effect of other *CO* genes.

In summary, around 75% of the *ppd-H1* accessions in this study originate from EU. EU spring barley accessions mainly carry *ppd-H1* alleles [9], thus leading to elongated post-AP phase durations due to the reduced response to LD. The collection used in this study, however, includes world-wide spring barley accessions which possess different genetic backgrounds, and hence, can easily explain the detected natural variation of heading time within this group. These results reinforce the importance of *CO-*like genes, specifically *HvCO1*, in the late-heading time pathway (*ppd-H1*) under LD.

Functional validation of candidate associations found in this work will help to complete our knowledge about developmental stages/sub-phase and/or heading time genetic networks in the future. Studying pre-anthesis development under GH and/or field conditions will greatly aid in the detection of causal variants in small grain cereals.

## Supporting Information

Figure S1 **Principal component analysis (PCA) of 218 spring barley accessions at heading stage using 6355 SNPs.** 95 spring barley accessions with photoperiod-sensitive (*Ppd-H1*) and 123 accessions with reduced photoperiod sensitivity (*ppd-H1*).
(TIF)

Figure S2 **Principal component analysis (PCA) of 218 spring barley accessions from different origins at heading stage using 6355 SNPs.** n = 45 for WANA, n = 108 for EU, n = 36 EA and n = 29 for AM.
(TIF)

Figure S3 **Manhattan plots of association findings.** The figures summarize GWAS obtained for heading date known gene (*Ppd-H1*) in whole spring barley collection using the iSelect 9K SNP platform. n = 218 accessions.
(TIF)

Figure S4 **Manhattan plots of association findings.** The figures summarize GWAS obtained from dissecting heading time at different stages in photoperiod-sensitive (*Ppd-H1*) and reduced photoperiod sensitivity (*ppd-H1*) barley accessions using the iSelect 9K SNP platform. Thermal time was taken from sowing to the beginning of awn primordium, tipping, heading and anther extrusion stages. The black dotted line marks the threshold significance levels (-log_10_ (*P-value*  = 0.01)), and SNPs in loci exceeding this threshold are considered as significantly associated.
(TIF)

Figure S5 **Manhattan plots of association findings.** The figures summarize GWAS obtained from dissecting heading time into sub-phases in photoperiod-sensitive (*Ppd-H1*) and reduced photoperiod sensitivity (*ppd-H1*) barley accessions using the iSelect 9K SNP platform. Thermal time was taken for the duration between sub-phases. The black dotted line marks the threshold significance levels (-log_10_ (*P-value*  = 0.01)), and SNPs in loci exceeding this threshold are considered as significantly associated.
(TIF)

Figure S6 **Number of significant SNPs for photoperiod-sensitive (** ***Ppd-H1*** **) and reduced photoperiod sensitivity (** ***ppd-H1*** **) at different developmental stages and sub-phases.** SNPs which have (-log_10_ >2, *P-value*  = 0.01) are considered as significant SNPs.
(TIF)

Figure S7 **False Discovery Rate (FDR) threshold (** ***P*** **= 0.05) at each developmental stage and sub-phase in barley accessions with photoperiod-sensitive (** ***Ppd-H1*** **) and reduced photoperiod sensitivity (** ***ppd-H1*** **).** SNPs exceeding FDR threshold are considered as highly significant SNPs.
(TIF)

Table S1 **Phenotypic data for developmental stages and sub-phases measured as thermal time ^○^C.D^−1^ (GDD) in the association mapping groups.**
(XLSX)

Table S2 **GenBank accession number for known heading time candidate genes with their POPSEQ genetic position and significantly associated markers (POPSEQ position in cM).**
(DOCX)

Table S3 **Number of QTL (within confidence interval ±5 cM) between developmental stages at each chromosome in groups with A) photoperiod-sensitive (** ***Ppd-H1*** **) and B) reduced photoperiod sensitivity (** ***ppd-H1*** **).** QTL exceeding threshold significance level (-log_10_ >2, *P*-value  = 0.01) are considered as significantly associated.
(DOCX)
