# Correction: Genetic Dissection of Photoperiod Response Based on GWAS of Pre-Anthesis Phase Duration in Spring Barley

**DOI:** 10.1371/journal.pone.0123748

**Published:** 2015-04-07

**Authors:** The PLOS ONE Staff

There are errors in the formatting of Table 1. Please see the correct Table 1 here.

**Table 1:** Spike row-type and origins of spring barley accessions with photoperiod-sensitive (*Ppd-H1)* and reduced photoperiod sensitivity (*ppd-H1*).

Origin^‡^	Photoperiod-sensitive(*Ppd-H1*)		Reduced photoperiod sensitivity (*ppd-H1*)		Total
	Two-rowed	Six-rowed	Two-rowed	Six-rowed	
**WANA**	12	21	11	1	45
**EU**	10	6	80	12	108
**EA**	0	28	2	6	36
**AM**	6	12	4	7	29
**Total**	**28**	**67**	**97**	**26**	**218**
	**95**		**123**		

^‡^ WANA: West Asia and North Africa, EU: Europe, EA: East Asia, AM, Americas.

The following errors appear in the body of the article.

In the second paragraph of the Introduction, *PSEUDO-RESPONSE REGULATOR (HvPRR37)* should be *PSEUDO-RESPONSE REGULATOR 37 (HvPRR37)*.

There are errors in the sixth sentence of the Materials and Methods subsection titled “Phenotyping.” The correct sentence is: To avoid any mineral deficiency each pot was additionally fertilized with 1.5 gram of solid fertilizer (that constitute minerals 17:11:10/N: P: K).

There are errors in the fourth sentence of the Results subsection titled “Identification of marker-trait association within the *ppd-H1*-carrying group.” The correct sentence is: However, *HvCO1* resides in a chromosomal region, which physically contains three other *CO*-like family members (*HvCO12/ HvCO13/HvM* and *HvCO15*) and the circadian clock-related genes, *HvLHY/HvCCA1*.

There are errors in the twelfth sentence of the fourth paragraph in the Discussion subsection titled “Heading time genetic network models.” The correct sentence is: Similar conclusions can be drawn from gene clusters on chromosome 4H, which besides *HvCO16* also includes *HvPRR59*, *HvPhyB* and *HvPrr73* (see 4H; 51.1–51.4 cM), 5HS (including *HvC)3*, *HvTFL1*, *HvCMF13;* 43.7–51.6 cM) and 1HS (*HvCMF10;* 41.1–48.2 cM).

There are errors in the fourteenth sentence of the fourth paragraph in the Discussion subsection titled “Heading time genetic network models.” The correct sentence is: Another group-specific effect was found for SNPs around the *FT-*like homolog *HvFT2* and the circadian clock-related gene *HvGI* (3HS; 45.8–52.0 cM).

There are errors in the third sentence of the sixth paragraph in the Discussion subsection titled “Heading time genetic network models.” The correct sentence is: Although its genetic position strongly suggests that the strongest associations reside very close to *HvCO1* (supported by 13 SNP markers; see Figure 9), we cannot completely rule out that linkage with other *CO* genes in this region like *HvCO12/ HvCO13/HvM* and *HvCO15* (Figure 7) contribute to this effect.
