# Supplementary figures and images for: Genetic Dissection of Photoperiod Response Based on GWAS of Pre-Anthesis Phase Duration in Spring Barley
Source: PLoS One. 2014 Nov 24;9(11):e113120. doi: 10.1371/journal.pone.0113120 (PMC4242610; doi:10.1371/journal.pone.0113120)

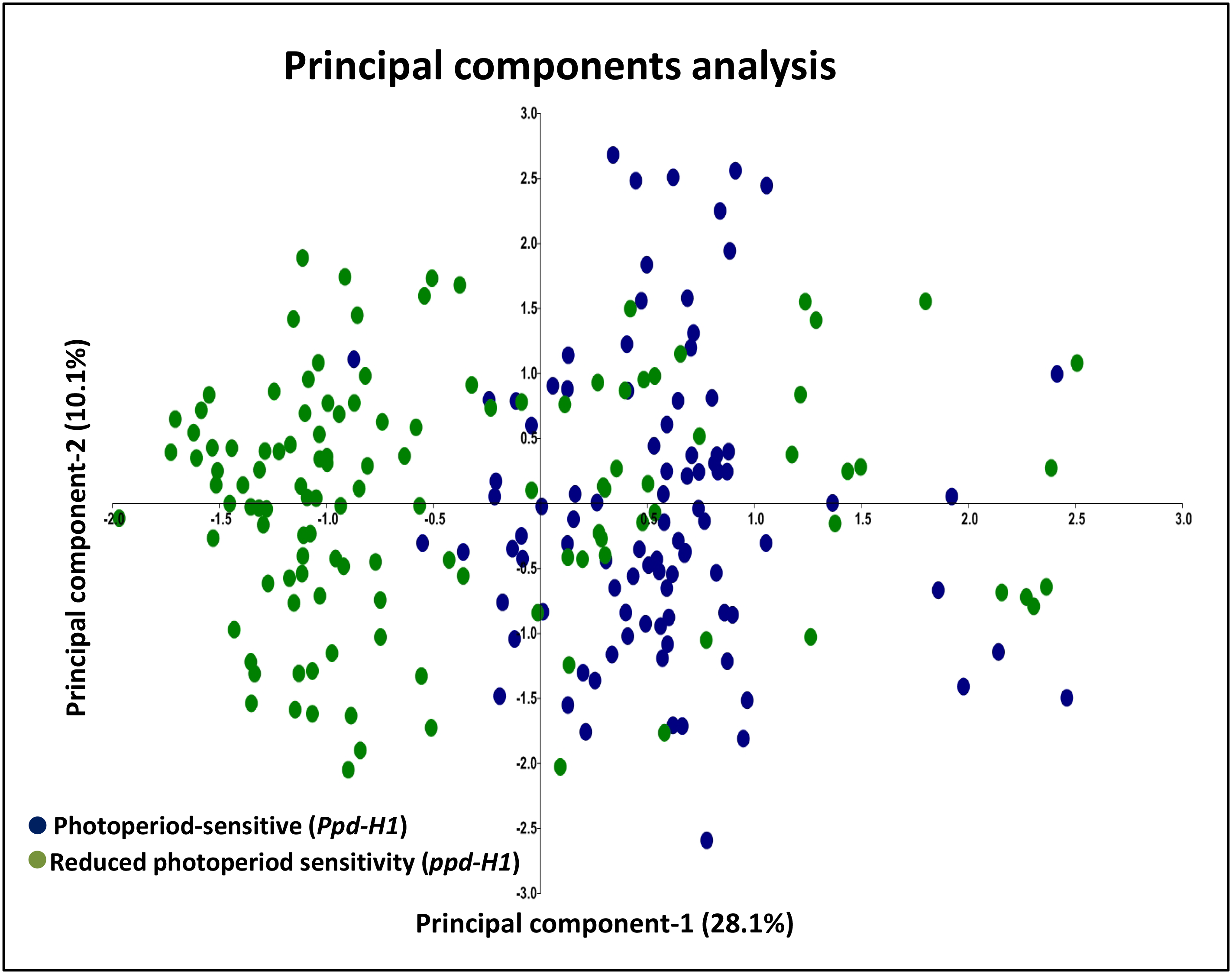

Supplement: Figure S1 — Principal component analysis (PCA) of 218 spring barley accessions at heading stage using 6355 SNPs. 95 spring barley accessions with photoperiod-sensitive (Ppd-H1) and 123 accessions with reduced photoperiod sensitivity (ppd-H1). (TIF) [file pone.0113120.s001.tif]

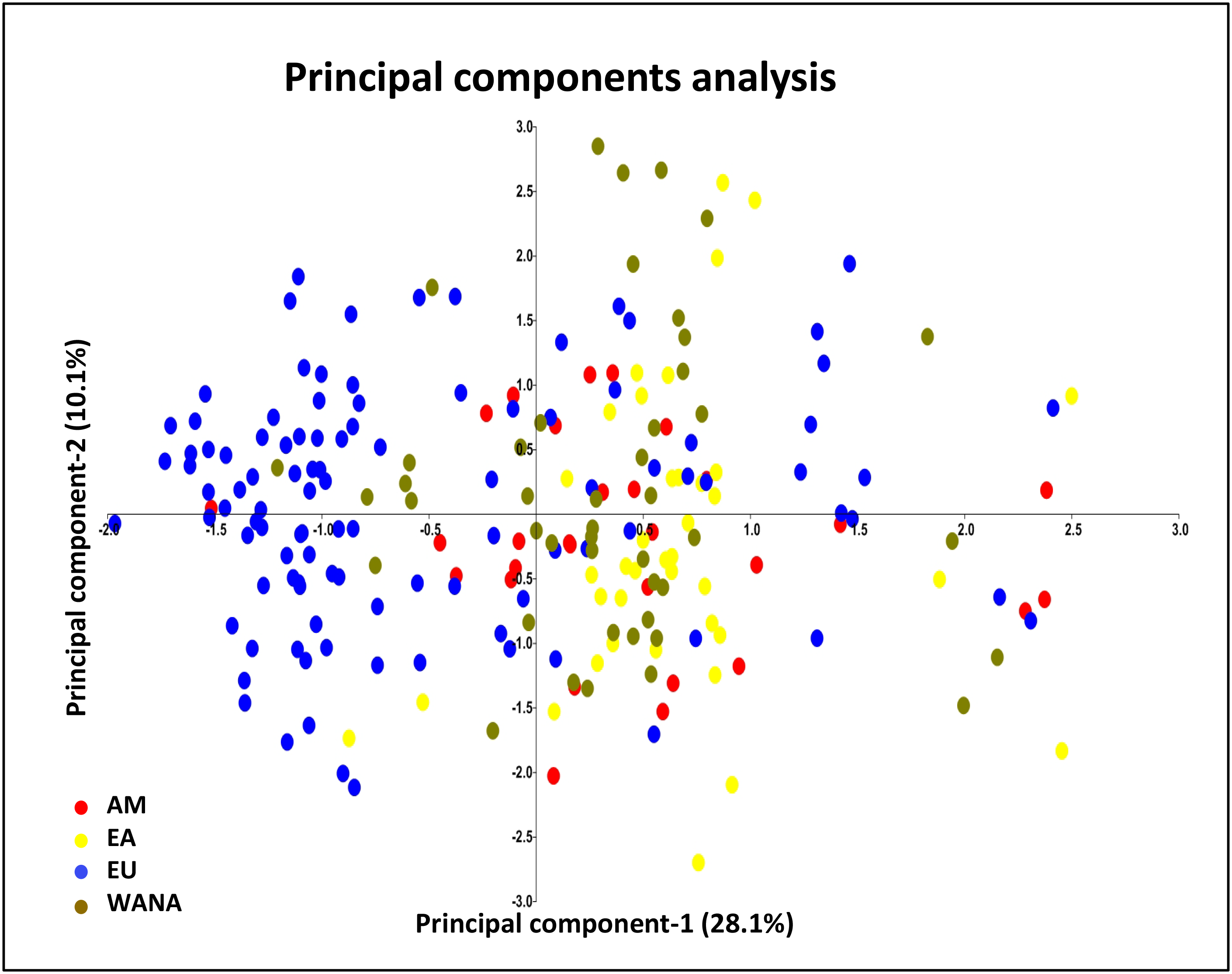

Supplement: Figure S2 — Principal component analysis (PCA) of 218 spring barley accessions from different origins at heading stage using 6355 SNPs. n = 45 for WANA, n = 108 for EU, n = 36 EA and n = 29 for AM. (TIF) [file pone.0113120.s002.tif]

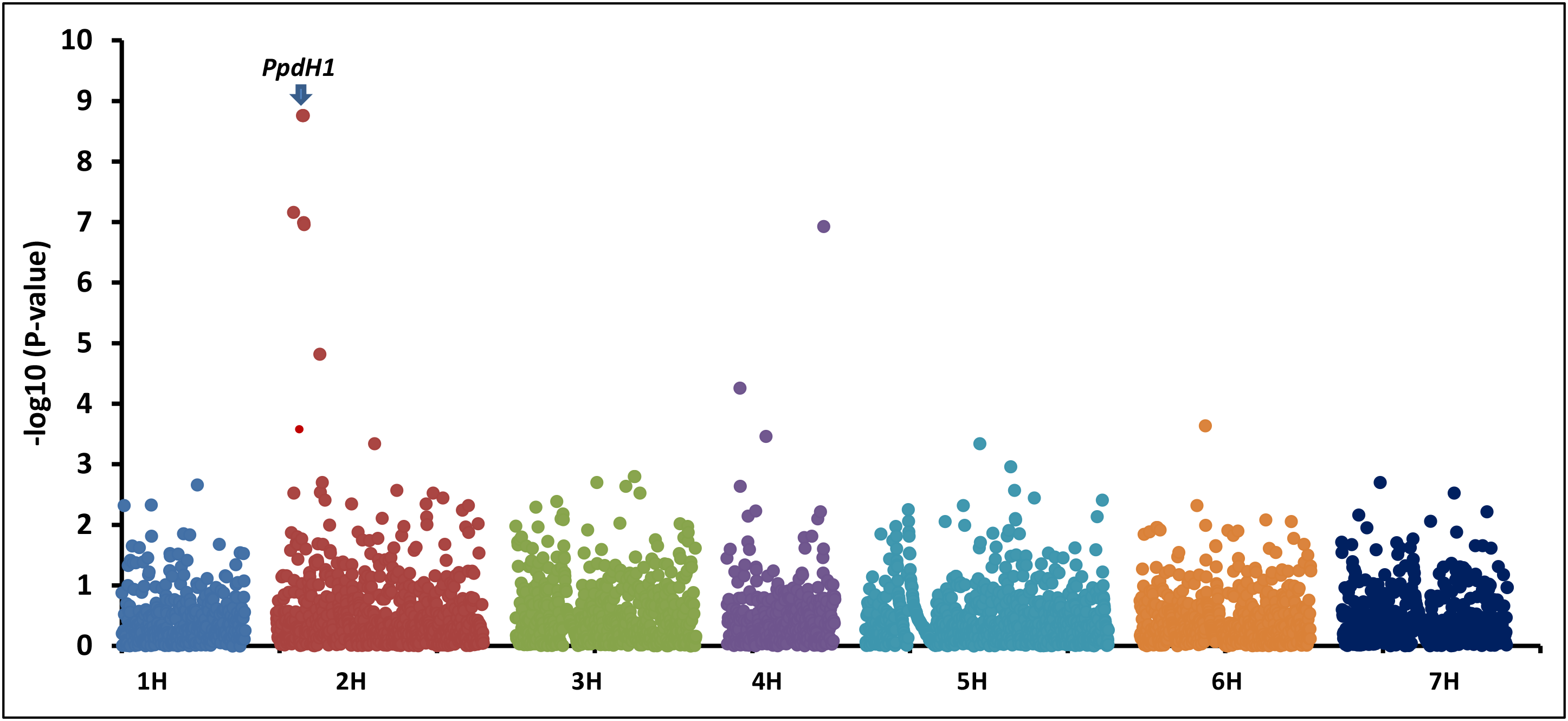

Supplement: Figure S3 — Manhattan plots of association findings. The figures summarize GWAS obtained for heading date known gene (Ppd-H1) in whole spring barley collection using the iSelect 9K SNP platform. n = 218 accessions. (TIF) [file pone.0113120.s003.tif]

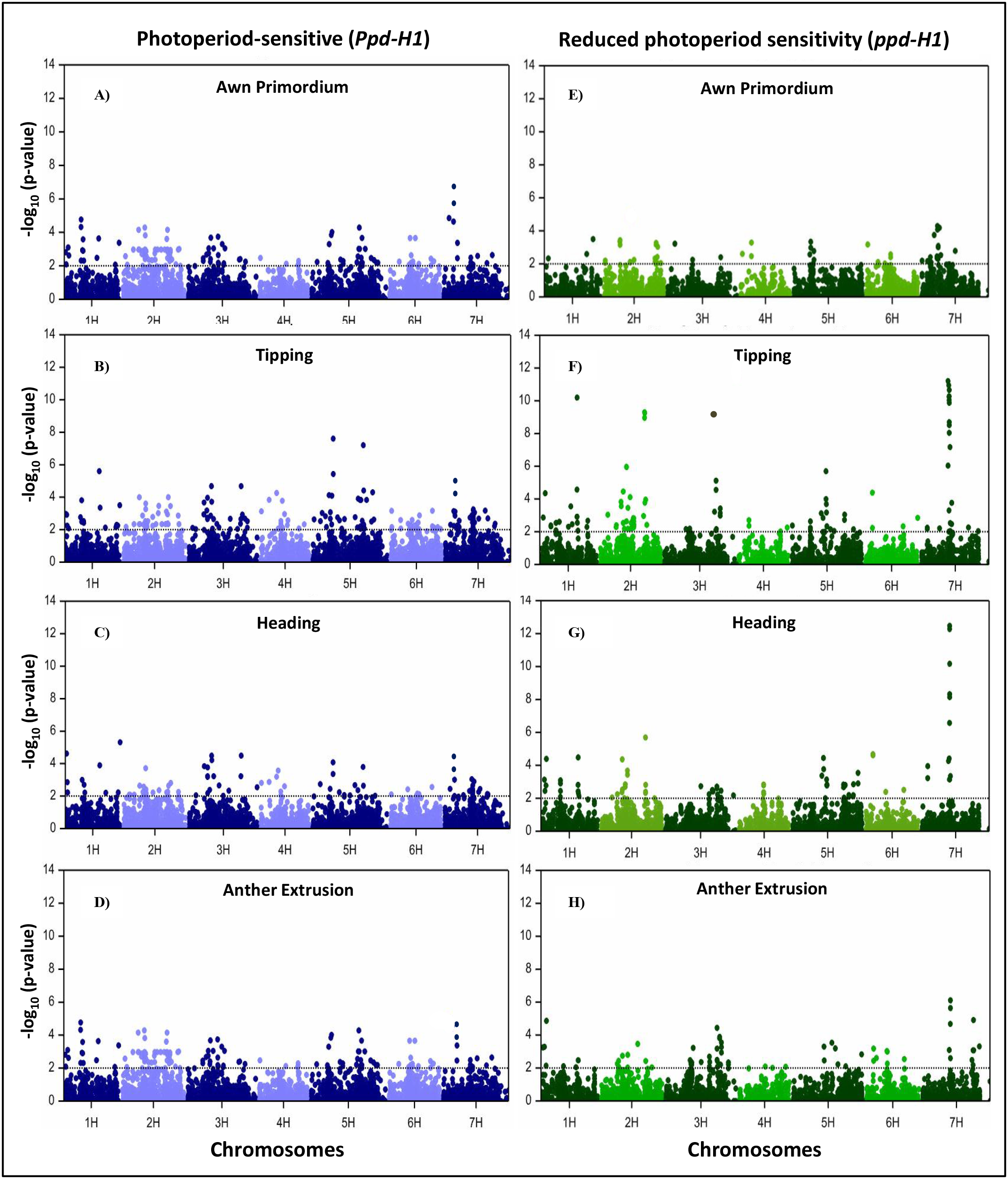

Supplement: Figure S4 — Manhattan plots of association findings. The figures summarize GWAS obtained from dissecting heading time at different stages in photoperiod-sensitive (Ppd-H1) and reduced photoperiod sensitivity (ppd-H1) barley accessions using the iSelect 9K SNP platform. Thermal time was taken from sowing to the beginning of awn primordium, tipping, heading and anther extrusion stages. The black dotted line marks the threshold significance levels (-log10 (P-value = 0.01)), and SNPs in loci exceeding this threshold are considered as significantly associated. (TIF) [file pone.0113120.s004.tif]

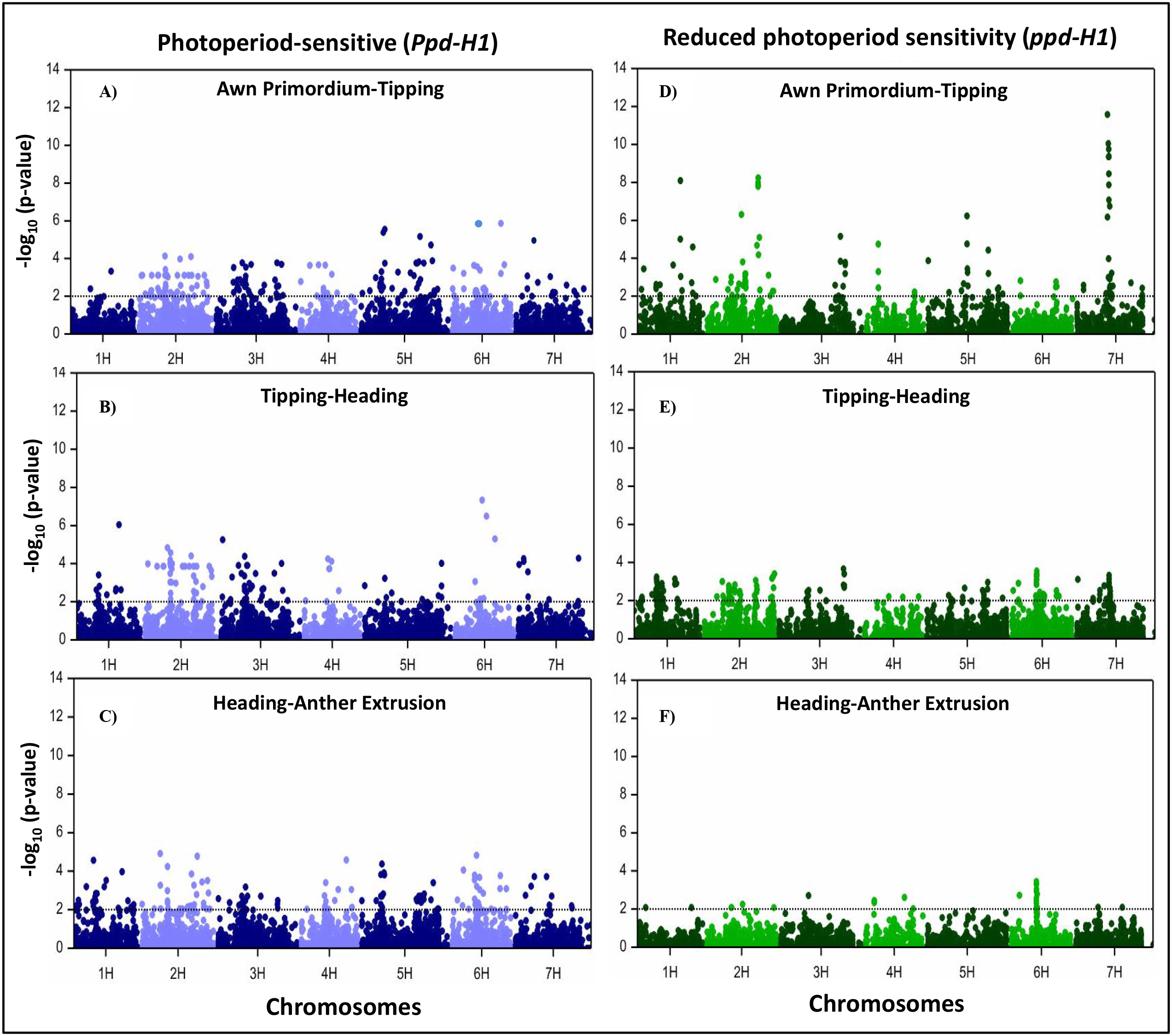

Supplement: Figure S5 — Manhattan plots of association findings. The figures summarize GWAS obtained from dissecting heading time into sub-phases in photoperiod-sensitive (Ppd-H1) and reduced photoperiod sensitivity (ppd-H1) barley accessions using the iSelect 9K SNP platform. Thermal time was taken for the duration between sub-phases. The black dotted line marks the threshold significance levels (-log10 (P-value = 0.01)), and SNPs in loci exceeding this threshold are considered as significantly associated. (TIF) [file pone.0113120.s005.tif]

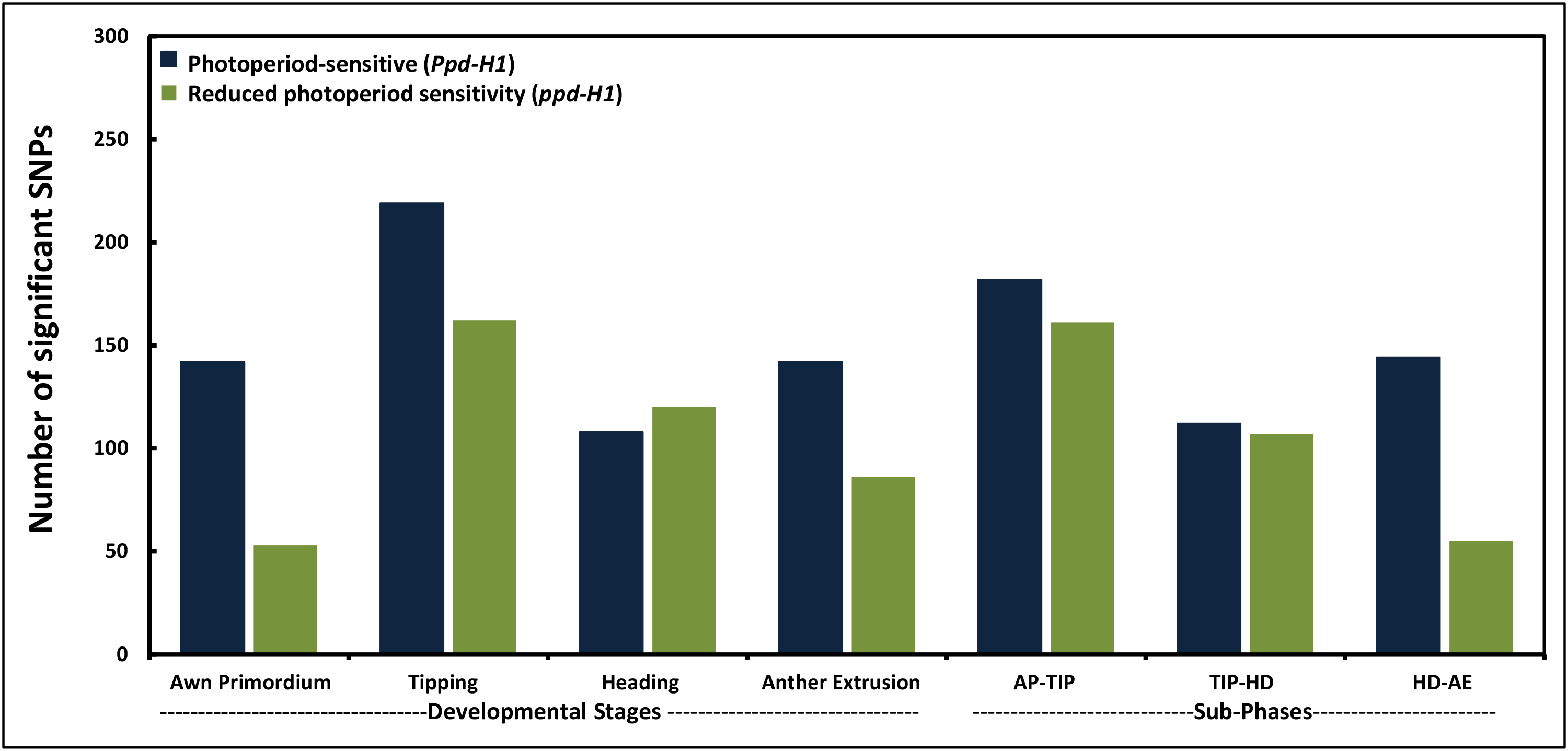

Supplement: Figure S6 — Number of significant SNPs for photoperiod-sensitive ( Ppd-H1 ) and reduced photoperiod sensitivity ( ppd-H1 ) at different developmental stages and sub-phases. SNPs which have (-log10 >2, P-value = 0.01) are considered as significant SNPs. (TIF) [file pone.0113120.s006.tif]

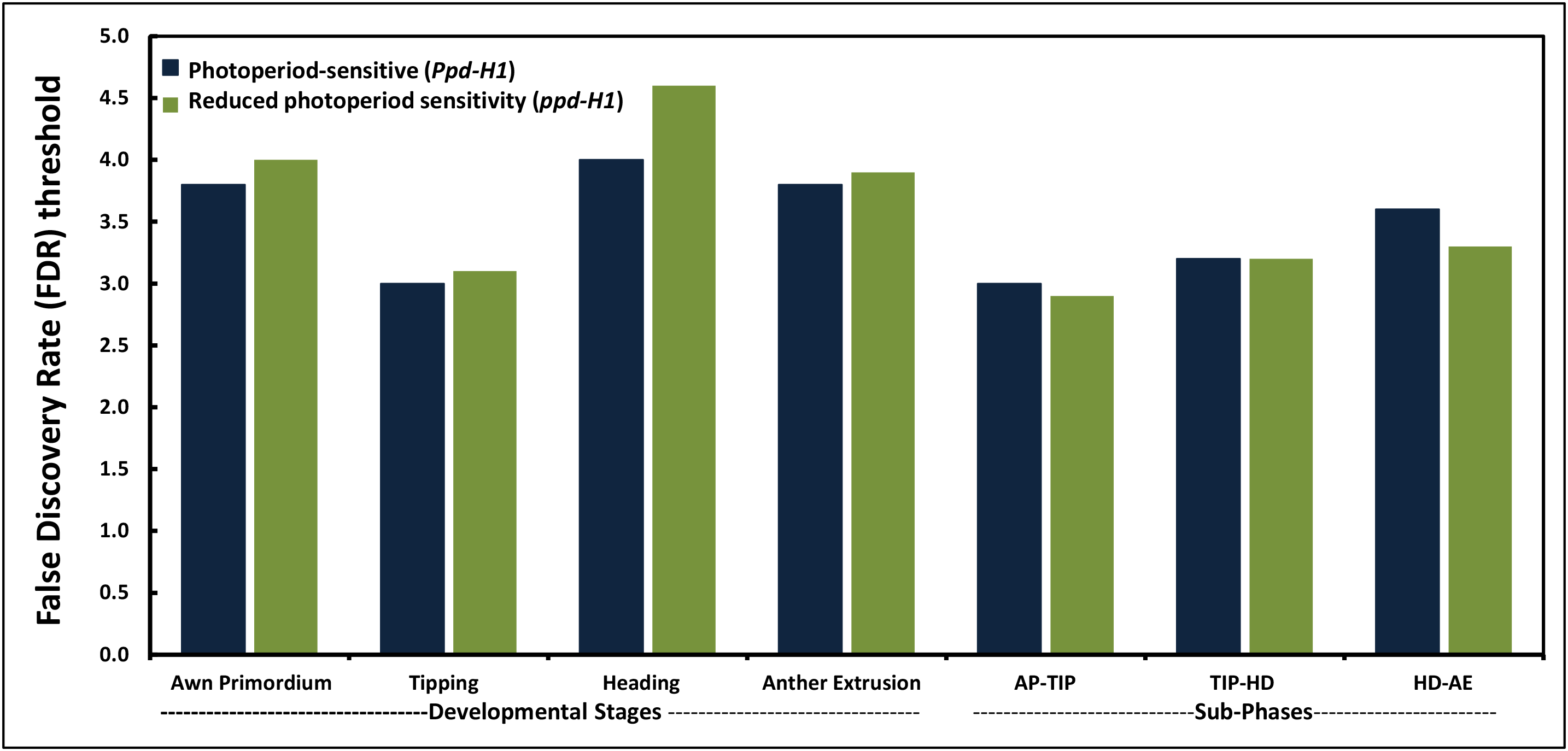

Supplement: Figure S7 — False Discovery Rate (FDR) threshold ( P = 0.05) at each developmental stage and sub-phase in barley accessions with photoperiod-sensitive ( Ppd-H1 ) and reduced photoperiod sensitivity ( ppd-H1 ). SNPs exceeding FDR threshold are considered as highly significant SNPs. (TIF) [file pone.0113120.s007.tif]
