# Supplementary material for: Genetic Dissection of Photoperiod Response Based on GWAS of Pre-Anthesis Phase Duration in Spring Barley
Source: PLoS One. 2014 Nov 24;9(11):e113120. doi: 10.1371/journal.pone.0113120 (PMC4242610; doi:10.1371/journal.pone.0113120)
Supplement: Table S2 — GenBank accession number for known heading time candidate genes with their POPSEQ genetic position and significantly associated markers (POPSEQ position in cM). (DOCX) [file pone.0113120.s009.docx]

**Table S2**: GenBank accession number for known heading time candidate genes with their POPSEQ genetic position and significantly associated markers (POPSEQ position in cM).

| **Chr.** | **Gene** | **Genebank accession number** | **Reference** | **cM (POP SEQ)** | **Contig identifier** | **Significantly associated**  **SNP marker**  **≥FDR (cM POPSEQ)** |
| --- | --- | --- | --- | --- | --- | --- |
| 1H | *HvCMF10* | [JQ791225](http://webblast.ipk-gatersleben.de/barley/blastresult.php?jobid=140120626639&opt=none) | [[1](#_ENREF_1)] | 47.82 | morex_contig_53826 CAJW010053826 | 12_30683 (46.6)  SCRI_RS_149971 (48.08) |
| 1H | *HvCO9/HvCMF11* | [AY082965](http://webblast.ipk-gatersleben.de/barley/blastresult.php?jobid=140120596769&opt=none) | [[2](#_ENREF_2),[3](#_ENREF_3)] | ~60*** | morex_contig_67944 CAJW010067944 |  |
| 1H | *HvCMF5* | [JQ791219](http://webblast.ipk-gatersleben.de/barley/blastresult.php?jobid=140120646281&opt=none) | [[1](#_ENREF_1)] | 81.72 | morex_contig_79857 CAJW010079857 |  |
| 1H | *Ppd-H2/HvFT3* | [HM133570.1](http://webblast.ipk-gatersleben.de/barley/blastresult.php?jobid=140120721998&opt=none) | [[4](#_ENREF_4)] | 92.35 | morex_contig_2551337 CAJW012551337 | SCRI_RS_138010 (92.06)  SCRI_RS_199689 (92.35) |
| 1H | *HvAdh2* | [AY184935.1](http://webblast.ipk-gatersleben.de/barley/blastresult.php?jobid=140120660621&opt=none) | [[5](#_ENREF_5)] | 104.8 | morex_contig_43476 CAJW010043476 | SCRI_RS_238125 (103.82) |
| 1H | *HvCMF6a* | [JQ791220](http://webblast.ipk-gatersleben.de/barley/blastresult.php?jobid=140120701589&opt=none) | [[1](#_ENREF_1)] | 132.02 | morex_contig_2548366 CAJW012548366 | SCRI_RS_153896 (132.08) |
| 1H | *Esp1L/HvELF3*  */eam8* | [JN180296.1](http://webblast.ipk-gatersleben.de/barley/blastresult.php?jobid=140164502833&opt=none#BL_ORD_ID:2822971) | [[6](#_ENREF_6)] | 132.36 | morex_contig_80895 CAJW010080895 | SCRI_RS_199945 (132.86) |
| 1H | *HvCMF6b* | [JQ791252](http://webblast.ipk-gatersleben.de/barley/blastresult.php?jobid=140120708529&opt=none) | [[1](#_ENREF_1)] | 132.57 | morex_contig_174751 CAJW010174751 | SCRI_RS_199945 (132.86) |
| 2H | *PpdH1* | [AY970701.1](http://webblast.ipk-gatersleben.de/barley/blastresult.php?jobid=140120777720&opt=none) | [[2](#_ENREF_2)] | 19.90 | morex_contig_94710 CAJW010094710 | BK_12 (19.90)  BK_13 (19.90)  BK_15 (19.90)  BK_16 (19.90) |
| 2H | *HvCO18* | [JQ791251](http://webblast.ipk-gatersleben.de/barley/blastresult.php?jobid=140120737651&opt=none) | [[1](#_ENREF_1)] | 41.85 | morex_contig_68173 CAJW010068173 | 12_10296 (40.08)  SCRI_RS_154981 (41.20) |
| 2H | *HvFT4* | [DQ411320.1](http://webblast.ipk-gatersleben.de/barley/blastresult.php?jobid=140120761128&opt=none) | [[7](#_ENREF_7)] | 50.04 | morex_contig_6666 CAJW010006666 | SCRI_RS_221843 (49.40) |
| 2H | *eps2/HvCEN/eam6* | [JX844786](http://webblast.ipk-gatersleben.de/barley/blastresult.php?jobid=140120748824&opt=none).1 | [[2](#_ENREF_2)] | 58.00 | morex_contig_274284 CAJW010274284 | 12_30634 (58.07)  11_10358 (58.71)  SCRI_RS_83731 (58.78) |
| 2H | *HD6-2H* | [DQ157464.1](http://webblast.ipk-gatersleben.de/barley/blastresult.php?jobid=140125780669&opt=none) | <http://kasetsartjournal.ku.ac.th/kuj_files/2008/A080402133514.pdf> | 59.41 | morex_contig_1567582 CAJW011567582 |  |
| 2H | *HvCO4* | [AF490474](http://webblast.ipk-gatersleben.de/barley/blastresult.php?jobid=140120756094&opt=none) | [[3](#_ENREF_3)] | 64.73 | morex_contig_161048 CAJW010161048 | SCRI_RS_59851 (64.58) |
| 2H | *BFL/HvAPO2* | [AB005620.1](http://webblast.ipk-gatersleben.de/barley/blastresult.php?jobid=140120769868&opt=none) | [[8](#_ENREF_8)] | 107.36 | morex_contig_1567741 CAJW011567741 | 11_10989 (107.93) |
| 2H | *HvAP2* | [GQ403050.1](http://webblast.ipk-gatersleben.de/barley/blastresult.php?jobid=140161648340&opt=none) | [[9](#_ENREF_9)] | 126.7 | morex_contig_46950 CAJW010046950 |  |
| 2H | *Ertr67 HvAP2*  *(Zeo)* | [KC898651](http://webblast.ipk-gatersleben.de/barley/blastresult.php?jobid=140161945454&opt=none#BL_ORD_ID:2785527) | [[10](#_ENREF_10)] | 127.05 | morex_contig_43451 CAJW010043451 |  |
| 3H | *HvGI* | [AY740524.1](http://webblast.ipk-gatersleben.de/barley/blastresult.php?jobid=140120805419&opt=none) | [[11](#_ENREF_11)] | 45.82 | morex_contig_58270 CAJW010058270 | 12_31475 (45.82) |
| 3H | *HvFT2* | [DQ297407](http://webblast.ipk-gatersleben.de/barley/blastresult.php?jobid=140120896646&opt=none) | [[7](#_ENREF_7)] | 52.03 | morex_contig_1558556 CAJW011558556 | 11_20325 (52.02) |
| 3H | *HvCMF1* | [JQ791213](http://webblast.ipk-gatersleben.de/barley/blastresult.php?jobid=140120922328&opt=none) | [[1](#_ENREF_1)] | 98.22 | morex_contig_43834 CAJW010043834 |  |
| 3H | *HvLUX/eps3L/ eam10* | [KC668273.1](http://webblast.ipk-gatersleben.de/barley/blastresult.php?jobid=140120951267&opt=none) | [[12](#_ENREF_12)] | 149.00 | morex_contig_2548416 CAJW012548416 |  |
| 4H | *Vrn-H2/ HvZCCT-Ha/b/c* | AK365195.1 | [[2](#_ENREF_2)] | 114.94‡ | morex_contig_2199658 CAJW012199658 | SCRI_RS_151357 (114.94) |
| 4H | *HvCO10* | [JQ791236](http://webblast.ipk-gatersleben.de/barley/blastresult.php?jobid=140120977259&opt=none).1 | [[1](#_ENREF_1)] | 26.34 | morex_contig_7813 CAJW010007813 | SCRI_RS_207768 (26.3) |
| 4H | *HvPhyA* | [DQ201141.1](http://webblast.ipk-gatersleben.de/barley/blastresult.php?jobid=140120997421&opt=none) | [[13](#_ENREF_13)] | 34.56 | morex_contig_9764 CAJW010009764 |  |
| 4H | *HvCO16* | [JQ791248.1](http://webblast.ipk-gatersleben.de/barley/blastresult.php?jobid=140121014470&opt=none) | [[1](#_ENREF_1)] | 51.13 | morex_contig_44067 CAJW010044067 | 11_21071 (50.99)  SCRI_RS_171142 (51.34) |
| 4H | *HvPRR59* | [JQ791228](http://webblast.ipk-gatersleben.de/barley/blastresult.php?jobid=140121038873&opt=none) | [[1](#_ENREF_1)] | 51.34 | morex_contig_46739 CAJW010046739 | 11_21071 (50.99)  SCRI_RS_171142 (51.34) |
| 4H | *HvphyB* | [DQ201142](http://webblast.ipk-gatersleben.de/barley/blastresult.php?jobid=140121063126&opt=none) | [[13](#_ENREF_13)] | 51.40 | morex_contig_1557904 CAJW011557904 | 11_21071 (50.99)  SCRI_RS_171142 (51.34) |
| 4H | *HvPRR73* | [JQ791230](http://webblast.ipk-gatersleben.de/barley/blastresult.php?jobid=140121052618&opt=none) | [[1](#_ENREF_1)] | 51.40 | morex_contig_1563982 CAJW01156398 | 11_21071 (50.99)  SCRI_RS_171142 (51.34) |
| 4H | *HvCMF4* | [JQ791217](http://webblast.ipk-gatersleben.de/barley/blastresult.php?jobid=140121079763&opt=none) | [[1](#_ENREF_1)] | 103.75 | morex_contig_135706 CAJW010135706 | SCRI_RS_179398 (103.75) |
| 4H | *HvSOC1* | [JN673265.1](http://webblast.ipk-gatersleben.de/barley/blastresult.php?jobid=140188282299&opt=none#BL_ORD_ID:2798602) | [[14](#_ENREF_14)] | 107.32 | barke_contig_1803142 CAJV011657847 | 12_30385 (107.36) |
| 5H | *HvCO3* | [AF490473](http://webblast.ipk-gatersleben.de/barley/blastresult.php?jobid=140125439686&opt=none) | [[3](#_ENREF_3)] | 43.76 | morex_contig_67117 CAJW010067117 | SCRI_RS_219574 (43.68) |
| 5H | *HvTFL1* | [DQ539338.1](http://www.ncbi.nlm.nih.gov/nuccore/DQ539338.1) | <http://www.ncbi.nlm.nih.gov/nuccore/DQ539338.1> | 44.09 | morex_contig_2522905 CAJW012522905 | i_11_20461 (43.95) |
| 5H | *HvCMF13* | [JQ791226](http://webblast.ipk-gatersleben.de/barley/blastresult.php?jobid=140125426653&opt=none).1 | [[1](#_ENREF_1)] | 46.45 | morex_contig_1558212 CAJW011558212 | 11_10840 (47.79) |
| 5H | *HvPRR95* | [JQ791233](http://webblast.ipk-gatersleben.de/barley/blastresult.php?jobid=140125596838&opt=none) | [[1](#_ENREF_1)] | 97.29 | morex_contig_41351 CAJW010041351 | SCRI_RS_171047 (97.29) |
| 5H | *HvPhyC* | [AB827939.1](http://webblast.ipk-gatersleben.de/barley/blastresult.php?jobid=140125703080&opt=none) | [[15](#_ENREF_15)] | 125.76 | morex_contig_106547 CAJW010106547 | SCRI_RS_217212 (125.76) |
| 5H | *Vrn-H1* | [EF591648.1](http://webblast.ipk-gatersleben.de/barley/blastresult.php?jobid=140204296440&opt=none#BL_ORD_ID:5294173) | [[16](#_ENREF_16)] | 125.76 | morex_contig_2552097 CAJW012552097 | SCRI_RS_217212 (125.76) |
| 5H | *HD6-5H* | [DQ157464.1](http://webblast.ipk-gatersleben.de/barley/blastresult.php?jobid=140125780669&opt=none) | <http://kasetsartjournal.ku.ac.th/kuj_files/2008/A080402133514.pdf> | 128.19 | morex_contig_40406 CAJW010040406 | SCRI_RS_214130 (129.44) |
| 6H | *HvCMF3* | [JQ791216](http://webblast.ipk-gatersleben.de/barley/blastresult.php?jobid=140125913497&opt=none) | [[1](#_ENREF_1)] | 49.22 | morex_contig_56141 CAJW010056141 |  |
| 6H | *HvCO7* | [AY082963](http://webblast.ipk-gatersleben.de/barley/blastresult.php?jobid=140125885789&opt=none) | [[3](#_ENREF_3)] | 52.62 | morex_contig_2550116 CAJW012550116 |  |
| 6H | *HvCO5* | [AY082958](http://webblast.ipk-gatersleben.de/barley/blastresult.php?jobid=140125935217&opt=none) | [[3](#_ENREF_3)] | 55.02 | morex_contig_243021 CAJW010243021 | 12_30857 (55.02) |
| 6H | *HvCry1a* | [DQ201149](http://webblast.ipk-gatersleben.de/barley/blastresult.php?jobid=140126090274&opt=none) | [[13](#_ENREF_13)] | 55.02 | morex_contig_75574 CAJW010075574 | 12_30857 (55.02) |
| 6H | *HvCry2* | [DQ201155](http://webblast.ipk-gatersleben.de/barley/blastresult.php?jobid=140126139716&opt=none#BL_ORD_ID:2883973) | [[13](#_ENREF_13)] | 55.02 | morex_contig_141897 CAJW010141897 | 12_30857 (55.02) |
| 6H | *HvPRR1/HvTOC1* | [JQ791234](http://webblast.ipk-gatersleben.de/barley/blastresult.php?jobid=140126006288&opt=none) | [[1](#_ENREF_1)] | 55.38 | morex_contig_37494 CAJW010037494 | SCRI_RS_237887 (55.10) |
| 6H | *HvCry1b* | [DQ201152](http://webblast.ipk-gatersleben.de/barley/blastresult.php?jobid=140126123570&opt=none) | [[13](#_ENREF_13)] | 59.06 | morex_contig_48345 CAJW010048345 | SCRI_RS_10810 (59.40) |
| 6H | *HvCO14* | [JQ791244](http://webblast.ipk-gatersleben.de/barley/blastresult.php?jobid=140126037462&opt=none) | [[1](#_ENREF_1)] | 67.91 | morex_contig_367999 CAJW010367999 | SCRI_RS_136724 (67.90) |
| 6H | *HvCO2* | [AF490469](http://webblast.ipk-gatersleben.de/barley/blastresult.php?jobid=140126056975&opt=none) | [[3](#_ENREF_3)] | 68.20 | morex_contig_6805 CAJW010006805 | SCRI_RS_165594 (62.74) |
| 6H | *HvCO11* | [JQ791238](http://webblast.ipk-gatersleben.de/barley/blastresult.php?jobid=140126074279&opt=none) | [[1](#_ENREF_1)] | 69.26 | morex_contig_1577721 CAJW011577721 | 11_20784 (69.05)  12_31250 (69.05) |
| 7H | *Vrn-H3/HvFT1* | [DQ100327](http://webblast.ipk-gatersleben.de/barley/blastresult.php?jobid=140190023192&opt=none#BL_ORD_ID:2797059) | [[2](#_ENREF_2)] | 34.43‡ | morex_contig_54983 CAJW010054983 | 12_30894 (34.34)  12_30895 (34.34) |
| 7H | *WAXY* | [AF486515.1](http://webblast.ipk-gatersleben.de/barley/blastresult.php?jobid=140126586059&opt=none) | [[17](#_ENREF_17)] | 13.88 | morex_contig_49158 CAJW010049158 | SCRI_RS_152931 (11.54) |
| 7H | *HvCO8* | [AY082964](http://webblast.ipk-gatersleben.de/barley/blastresult.php?jobid=140126852493&opt=none#BL_ORD_ID:3110845) | [[3](#_ENREF_3)] | 41.99 | morex_contig_368769 CAJW010368769 | SCRI_RS_187827 (43.83) |
| 7H | *HvSS1* | [FN400939.1](http://webblast.ipk-gatersleben.de/barley/blastresult.php?jobid=140126926482&opt=none#BL_ORD_ID:4303873) | <http://www.ncbi.nlm.nih.gov/nuccore/239984689> | 54.39 | morex_contig_1561797 CAJW011561797 |  |
| 7H | *HvVRT-2* | [AK355370](http://webblast.ipk-gatersleben.de/barley/blastresult.php?jobid=140126820430&opt=none#BL_ORD_ID:2779415) | [[13](#_ENREF_13)] | 61.75 | morex_contig_37339 CAJW010037339 | 11_20975 (61.75) |
| 7H | *HvCO12* | [JQ791240](http://webblast.ipk-gatersleben.de/barley/blastresult.php?jobid=140126874462&opt=none#BL_ORD_ID:2882314) | [[1](#_ENREF_1)] | 67.77 | morex_contig_140238 CAJW010140238 | SCRI_RS_235641 (67.77) |
| 7H | *HvCO13/HvM* | [JQ791242](http://webblast.ipk-gatersleben.de/barley/blastresult.php?jobid=140126885490&opt=none#BL_ORD_ID:2788072) | [[1](#_ENREF_1)] | 67.77 | morex_contig_45996 CAJW010045996 | SCRI_RS_235641 (67.77) |
| 7H | *HvCO1* | [AF490468.1](http://webblast.ipk-gatersleben.de/barley/blastresult.php?jobid=140126894698&opt=none#BL_ORD_ID:2880410) | [[3](#_ENREF_3)] | 67.91 | morex_contig_138334 CAJW010138334 | BK_03 (67.91)  12_31452 (67.91)  12_31452 (67.91)  SCRI_RS_108830 12_31452 (67.91)  SCRI_RS_124819 12_31452 (67.91)  SCRI_RS_127040 12_31452 (67.91)  SCRI_RS_132722 12_31452 (67.91)  SCRI_RS_152696 12_31452 (67.91)  SCRI_RS_171588 12_31452 (67.91)  SCRI_RS_171786 12_31452 (67.91)  SCRI_RS_192587 12_31452 (67.91)  SCRI_RS_198541 12_31452 (67.91)  SCRI_RS_207354 12_31452 (67.91)  SCRI_RS_219081 12_31452 (67.91) |
| 7H | *HvCO15* | [JQ791246](http://webblast.ipk-gatersleben.de/barley/blastresult.php?jobid=140127162385&opt=none#BL_ORD_ID:2801195) | [[1](#_ENREF_1)] | 70.53 | morex_contig_59119 CAJW010059119 | 11_10924 (70.53)  11_20828 (70.53)  12_30053 (70.67) |
| 7H | *HvCCA1* | [HQ850271.1](http://webblast.ipk-gatersleben.de/barley/blastresult.php?jobid=140162611876&opt=none#BL_ORD_ID:4309371) | [[18](#_ENREF_18)] | 70.82 | morex_contig_1567295 CAJW011567295 | 12_30053 (70.67)  SCRI_RS_200107 (70.83) |
| 7H | *HvLHY* | [HQ222606.1](http://webblast.ipk-gatersleben.de/barley/blastresult.php?jobid=140127006297&opt=none#BL_ORD_ID:4309371) | <http://www.uniprot.org/uniprot/E9M5R6> | 70.82 | morex_contig_1567295 CAJW011567295 | 12_30053 (70.67)  SCRI_RS_200107 (70.83) |
| 7H | *HvCMF7* | [JQ791222](http://webblast.ipk-gatersleben.de/barley/blastresult.php?jobid=140127179796&opt=none#BL_ORD_ID:184177) | [[1](#_ENREF_1)] | 91.78 | morex_contig_104939 CAJW010104939 |  |
| 7H | *HvCO6* | [AY082960](http://webblast.ipk-gatersleben.de/barley/blastresult.php?jobid=140127189716&opt=none#BL_ORD_ID:2749481) | [[3](#_ENREF_3)] | 120.82 | morex_contig_7405 CAJW010007405 |  |

* Based on genebank accession number we detected the contig identifier without POPSEQ position but Cockram *et al.* [[1](#_ENREF_1)] mapped it at around 60 cM on 1H.

‡ The position of these genes is based on genetic marker positions published in [[2](#_ENREF_2)].

1. Cockram J, Thiel T, Steuernagel B, Stein N, Taudien S, et al. (2012) Genome dynamics explain the evolution of flowering time CCT domain gene families in the Poaceae. PloS one 7: e45307.

2. Comadran J, Kilian B, Russell J, Ramsay L, Stein N, et al. (2012) Natural variation in a homolog of *Antirrhinum CENTRORADIALIS* contributed to spring growth habit and environmental adaptation in cultivated barley. Nature genetics 44: 1388-1392.

3. Griffiths S, Dunford RP, Coupland G, Laurie DA (2003) The evolution of CONSTANS-like gene families in barley, rice, and Arabidopsis. Plant Physiology 131: 1855-1867.

4. Casao MC, Igartua E, Karsai I, Lasa JM, Gracia MP, et al. (2011) Expression analysis of vernalization and day-length response genes in barley (*Hordeum vulgare* L.) indicates that *VRNH2* is a repressor of *PPDH2* (*HvFT3*) under long days. Journal of Experimental Botany 62: 1939-1949.

5. Lin J-Z, Morrell PL, Clegg MT (2002) The Influence of Linkage and Inbreeding on Patterns of Nucleotide Sequence Diversity at Duplicate Alcohol Dehydrogenase Loci in Wild Barley (Hordeum vulgare ssp. spontaneum). Genetics 162: 2007-2015.

6. Zakhrabekova S, Gough SP, Braumann I, Müller AH, Lundqvist J, et al. (2012) Induced mutations in circadian clock regulator Mat-a facilitated short-season adaptation and range extension in cultivated barley. Proceedings of the National Academy of Sciences 10.1073/pnas.1113009109.

7. Faure S, Higgins J, Turner A, Laurie DA (2007) The *FLOWERING LOCUS T*-like gene family in barley (*Hordeum vulgare*). Genetics 176: 599-609.

8. Kyozuka J, Konishi S, Nemoto K, Izawa T, Shimamoto K (1998) Down-regulation of RFL, the FLO/LFY homolog of rice, accompanied with panicle branch initiation. Proceedings of the National Academy of Sciences 95: 1979-1982.

9. Nair SK, Wang N, Turuspekov Y, Pourkheirandish M, Sinsuwongwat S, et al. (2010) Cleistogamous flowering in barley arises from the suppression of microRNA-guided HvAP2 mRNA cleavage. Proceedings of the National Academy of Sciences of the United States of America 107: 490-495.

10. Houston K, McKim SM, Comadran J, Bonar N, Druka I, et al. (2013) Variation in the interaction between alleles of HvAPETALA2 and microRNA172 determines the density of grains on the barley inflorescence. Proceedings of the National Academy of Sciences 110: 16675-16680.

11. Dunford RP, Griffiths S, Christodoulou V, Laurie DA (2005) Characterisation of a barley (Hordeum vulgare L.) homologue of the Arabidopsis flowering time regulator GIGANTEA. Theoretical and Applied Genetics 110: 925-931.

12. Gawronski P, Ariyadasa R, Himmelbach A, Poursarebani N, Kilian B, et al. (2014) A distorted circadian clock causes early flowering and temperature-dependent variation in spike development in the *Eps*-*3A^m^* mutant of einkorn wheat. Genetics 10.1534/genetics.113.158444.

13. Szűcs P, Karsai I, Zitzewitz J, Mészáros K, Cooper LLD, et al. (2006) Positional relationships between photoperiod response QTL and photoreceptor and vernalization genes in barley. Theoretical and Applied Genetics 112: 1277-1285.

14. Papaefthimiou D, Kapazoglou A, Tsaftaris AS (2012) Cloning and characterization of SOC1 homologs in barley (Hordeum vulgare) and their expression during seed development and in response to vernalization. Physiologia Plantarum 146: 71-85.

15. Nishida H, Ishihara D, Ishii M, Kaneko T, Kawahigashi H, et al. (2013) *Phytochrome* C is a key factor controlling long-day flowering in barley. Plant Physiology 163: 804-814.

16. Cockram J, Chiapparino E, Taylor SA, Stamati K, Donini P, et al. (2007) Haplotype analysis of vernalization loci in European barley germplasm reveals novel *VRN-H1* alleles and a predominant winter *VRN-H1/VRN-H2* multi-locus haplotype. TAG Theoretical and applied genetics Theoretische und angewandte Genetik 115: 993-1001.

17. Patron NJ, Smith AM, Fahy BF, Hylton CM, Naldrett MJ, et al. (2002) The Altered Pattern of Amylose Accumulation in the Endosperm of Low-Amylose Barley Cultivars Is Attributable to a Single Mutant Allele of Granule-Bound Starch Synthase I with a Deletion in the 5′-Non-Coding Region. Plant Physiology 130: 190-198.

18. Faure S, Turner AS, Gruszka D, Christodoulou V, Davis SJ, et al. (2012) Mutation at the circadian clock gene EARLY MATURITY 8 adapts domesticated barley (Hordeum vulgare) to short growing seasons. Proceedings of the National Academy of Sciences 10.1073/pnas.1120496109.
