# Supplementary material for: Genetic Dissection of Photoperiod Response Based on GWAS of Pre-Anthesis Phase Duration in Spring Barley
Source: PLoS One. 2014 Nov 24;9(11):e113120. doi: 10.1371/journal.pone.0113120 (PMC4242610; doi:10.1371/journal.pone.0113120)
Supplement: Table S3 — Number of QTL (within confidence interval ±5 cM) between developmental stages at each chromosome in groups with A) photoperiod-sensitive ( Ppd-H1 ) and B) reduced photoperiod sensitivity ( ppd-H1 ). QTL exceeding threshold significance level (-log10 >2, P-value = 0.01) are considered as significantly associated. (DOCX) [file pone.0113120.s010.docx]

**Table S3**: Number of QTL (within confidence interval ±5 cM) between developmental stages at each chromosome in groups with A) photoperiod-sensitive (*Ppd-H1*) and B) reduced photoperiod sensitivity (*ppd-H1*). QTL exceeding threshold significance level(-log_10_>2, *P*-value = 0.01) are considered as significantly associated.

**A) photoperiod-sensitive (*Ppd-H1*)**


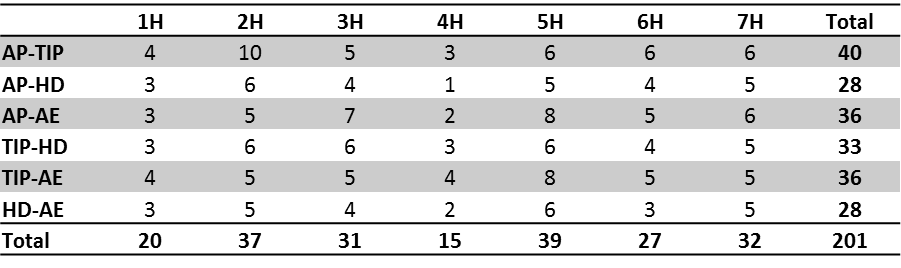


**B) Reduced photoperiod sensitivity (*ppd-H1*)**


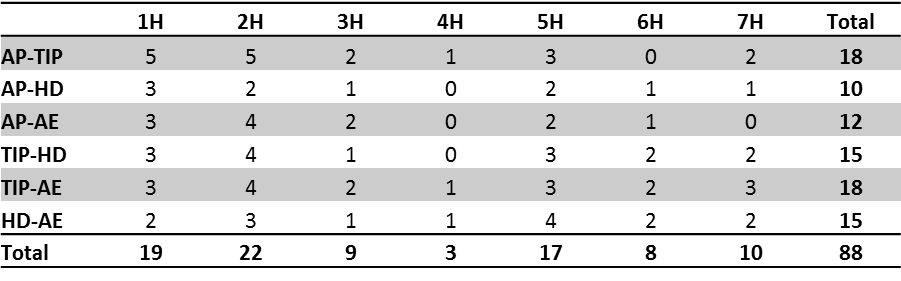


†AP: awn primordium, [3]; TIP: tipping, Z49; HD: heading, Z55; AE: anther extrusion, Z65, [32].
